# Regulatory changes associated with the head to trunk developmental transition

**DOI:** 10.1186/s12915-023-01675-2

**Published:** 2023-08-08

**Authors:** Patrícia Duarte, Rion Brattig Correia, Ana Nóvoa, Moisés Mallo

**Affiliations:** Instituto Gulbenkian de Ciência, Rua da Quinta Grande 6, 2780-156 Oeiras, Portugal

**Keywords:** Head to trunk transition, RNA-seq, ATAC-seq, Wnt5a, Retinoic acid, Nr2f2

## Abstract

**Background:**

Development of vertebrate embryos is characterized by early formation of the anterior tissues followed by the sequential extension of the axis at their posterior end to build the trunk and tail structures, first by the activity of the primitive streak and then of the tail bud. Embryological, molecular and genetic data indicate that head and trunk development are significantly different, suggesting that the transition into the trunk formation stage involves major changes in regulatory gene networks.

**Results:**

We explored those regulatory changes by generating differential interaction networks and chromatin accessibility profiles from the posterior epiblast region of mouse embryos at embryonic day (E)7.5 and E8.5. We observed changes in various cell processes, including several signaling pathways, ubiquitination machinery, ion dynamics and metabolic processes involving lipids that could contribute to the functional switch in the progenitor region of the embryo. We further explored the functional impact of changes observed in Wnt signaling associated processes, revealing a switch in the functional relevance of Wnt molecule palmitoleoylation, essential during gastrulation but becoming differentially required for the control of axial extension and progenitor differentiation processes during trunk formation. We also found substantial changes in chromatin accessibility at the two developmental stages, mostly mapping to intergenic regions and presenting differential footprinting profiles to several key transcription factors, indicating a significant switch in the regulatory elements controlling head or trunk development. Those chromatin changes are largely independent of retinoic acid, despite the key role of this factor in the transition to trunk development. We also tested the functional relevance of potential enhancers identified in the accessibility assays that reproduced the expression profiles of genes involved in the transition. Deletion of these regions by genome editing had limited effect on the expression of those genes, suggesting the existence of redundant enhancers that guarantee robust expression patterns.

**Conclusions:**

This work provides a global view of the regulatory changes controlling the switch into the axial extension phase of vertebrate embryonic development. It also revealed mechanisms by which the cellular context influences the activity of regulatory factors, channeling them to implement one of several possible biological outputs.

**Supplementary Information:**

The online version contains supplementary material available at 10.1186/s12915-023-01675-2.

## Background

During embryonic development the vertebrate body is generated progressively in a head to tail sequence. Although this is a continuous process it occurs in three distinct steps that produce head, trunk and tail structures [1–3]. Each of these stages is characterized by distinct cell dynamics and the generation of a specific set of tissues. For instance, during head development, the embryo establishes the main body axis, lays down the anlage for future brain structures and engages in the process of gastrulation to generate the germ layers [1, 4]. The latter process requires the induction of the primitive streak at the posterior end of the embryo that organizes the emergence of the embryonic endoderm as well as the mesodermal tissues for the head and heart primordia [4]. Genetic analyses in mice have identified key regulators involved in these processes. Some examples include interactions between *Nodal*, *Bmp4* and *Wnt3* to form the primitive streak [5], *Eomes* for the specification of the endodermal layer and mesoderm delamination [6], and *Gata4* and *Gata6* for heart induction [7]. The switch to trunk development is associated with major changes in the growth dynamics of the embryo. It starts elongating the main body axis at the posterior embryonic end by the progressive addition of new tissue produced by the activity of axial progenitors [2, 3, 8]. This process is associated with the emergence of the neuro-mesodermal competent (NMC) population, the progenitor cells that build the spinal cord and the axial skeleton [2, 8–10]. Additional progenitors in the epiblast also lay down the tissues that will contribute to the formation and vascularization of the organs involved in digestive, excretory and reproductive functions of the animal [11]. Similarly to the cells contributing to most embryonic tissues during head development, the progenitors generating trunk structures are also part of the epiblast, which at this stage occupies the posterior end of the embryo [2, 9, 12]. Also, the primitive streak keeps being the main organizer of progenitor activity during trunk development [2, 8]. However, the regulatory processes undergo major changes. Inactivation of *Tbxt*, the *Cdx* genes, *Wnt3a*, and the combined *Wnt5a* and *Wnt11* loss of function results in embryo truncation at the head to trunk transition, indicating their essential role for trunk development [13–19]. Other factors, like retinoic acid (RA), known to play essential roles during early stages of brain and heart development [20], are also required for trunk development, as silencing this signaling, most typically through inactivation of *Raldh2*, results in developmental arrest at the head to trunk transition [21]. However, the role of RA in this process might differ from that of the other factors, since axial extension can proceed in the absence of this signaling provided that the transition to trunk development is rescued by an acute exogenous RA administration [22].

These observations indicate that the transition into trunk development is associated with a global change in gene regulatory networks, most particularly in the posterior region of the embryo, that switches from gastrulation movements to axial extension. Importantly, many of the factors that control developmental processes during trunk extension are also expressed at earlier stages of development, despite not being required at those stages according to genetic experiments. This indicates that the head to trunk transition also involves a change in the capacity of cells to respond to regulatory factors when entering the trunk formation stage. From a regulatory perspective, this might involve modification of transcription factor (TF) accessibility to their functional targets in the genome. Recent studies using single cell approaches have mapped the molecular events involved in the formation of the major embryonic lineages during early organogenesis in mouse embryos [23, 24]. However, the regulatory transition from head to trunk formation has yet to be addressed.

In this study, we aimed to understand the mechanisms involved in the switch from head to trunk development. For this, we compared transcriptome and chromatin accessibility profiles from the posterior epiblast region of wild type mouse embryos at embryonic day (E)7.5 and E8.5. We observed significant changes in transcriptomic profiles between these two stages. In addition to the expected changes in factors involved in pluripotency and in the *Hox* gene profiles, we observed modifications in a variety of functional groups, including Wnt signaling pathway, ubiquitination systems and lipid metabolic profiles that might interact together to change functional properties at the progenitor region of the embryo. We also observed major changes in chromatin accessibility profiles mostly involving intergenic regions, thus indicating a major switch in regulatory elements controlling head or trunk development, which were associated with changes in the binding activity of key transcription factors. We also found that the absence of RA activity has very limited impact on the changes in chromatin accessibility. In addition, we performed functional tests on specific enhancers identified in the chromatin analyses, including potential regulators of *Wnt5a* and *Nr2f2.* In transgenic reporter experiments these enhancers showed activity compatible with the regulation of the candidate target genes. However, when removed from the genome by edition procedures they had very limited effect on the expression of those genes, indicating the existence of redundant enhancers that provide robustness to the system.

## Results & discussion

### Transcriptome profile of the posterior epiblast in the developing embryo

**Fig. 1:**
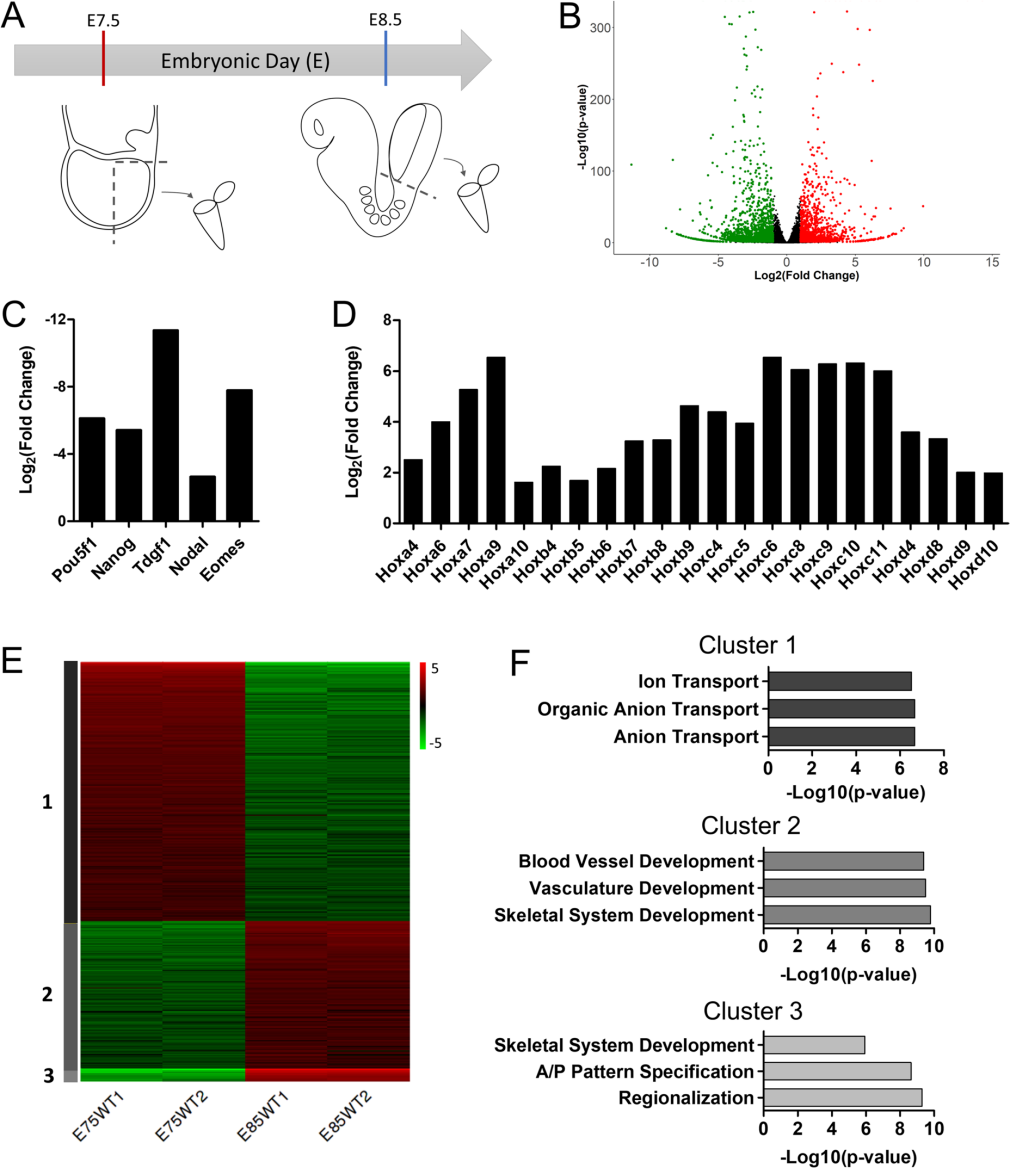
Transcriptomic changes in the posterior epiblast associated with the head to trunk transition. **A** Schematic representation of sample collection from the posterior epiblast region of mouse embryos at E7.5 and E8.5. **B** Volcano plot of RNA-seq gene expression (|Log_2_(Fold Change)|≥ 1 & *p*-value < 0.05). Significantly upregulated genes at E8.5 are in red, downregulated at E8.5 are in green and non-significant in black. **C**-**D** Gene expression of key pluripotent and early developmental genes (**C**) and Hox genes (**D**). **E** K-means clustering of the 1000 most variable genes. Cluster 1: 616 genes; Cluster 2: 352 genes; Cluster 3: 32 genes. **F** Top 3 GO terms from biological processes associated with Cluster 1, 2 and 3

To explore the changes in expression of genes involved in trunk formation, we used RNA-seq to obtain the transcriptome profiles from the posterior epiblast region of wild type mouse embryos at E7.5 and E8.5 (Fig. 1A), representing respectively the progenitor-containing region of embryos before and after they engage in trunk formation. Principal component analysis separated the samples by timepoint (Additional file 1: Fig. S1A), revealing the presence of distinct transcriptomic profiles at these two developmental stages. Differential analysis revealed the presence of 2090 genes significantly downregulated, and 1668 genes upregulated at E8.5 relative to E7.5 (Fig. 1B and Additional file 2: Table S1). Manual inspection of the list of differentially expressed genes (DEGs) identified downregulation at E8.5 of pluripotency genes, like *Pou5f1* or *Nanog* [25, 26], and genes involved in the initial establishment of the body axis and germ layers like *Tdgf1* (*Cripto*), *Nodal* or *Eomes* (Fig. 1C) [6, 27, 28]. Conversely, activation of central and posterior *Hox* genes was clearly observed at E8.5 (Fig. 1D). These findings fit with expression patterns reported for these genes, thus serving as an initial validation of our approach.

K-means clustering of the top 1000 most variable genes produced three clusters with distinct gene expression dynamics (Fig. 1E, F). Cluster 1 includes genes that became downregulated at E8.5; genes in this cluster are enriched in gene ontology (GO) terms related to anion and ion transport. Interestingly, a similar decrease in expression of genes enriched for ion transport and homeostasis has been described at the whole embryo level during the same stages analyzed here [29], further suggesting an important role for changes in ion transport profiles during early embryonic development. The full implication of this finding remains elusive. The control of ion fluxes has been implicated in patterning processes [30, 31], including early stages in the establishment of left–right asymmetry associated with node activity [32, 33]. They also have been shown to control cell processes involved in cell migration, cell proliferation and autophagy [34–36]. Focused experimental approaches will be required to explore if the drastic changes in ion transporter profiles observed in the progenitor-containing region during the head to trunk transition play a relevant role in the transition. Cluster 2 comprises genes moderately upregulated at E8.5, mostly associated with skeletal system, vasculature, and blood vessel development. Finally, cluster 3 is composed of genes strongly upregulated at E8.5. Genes in this cluster are enriched in skeletal system development, anterior/posterior pattern specification, and regionalization.

**Fig. 2:**
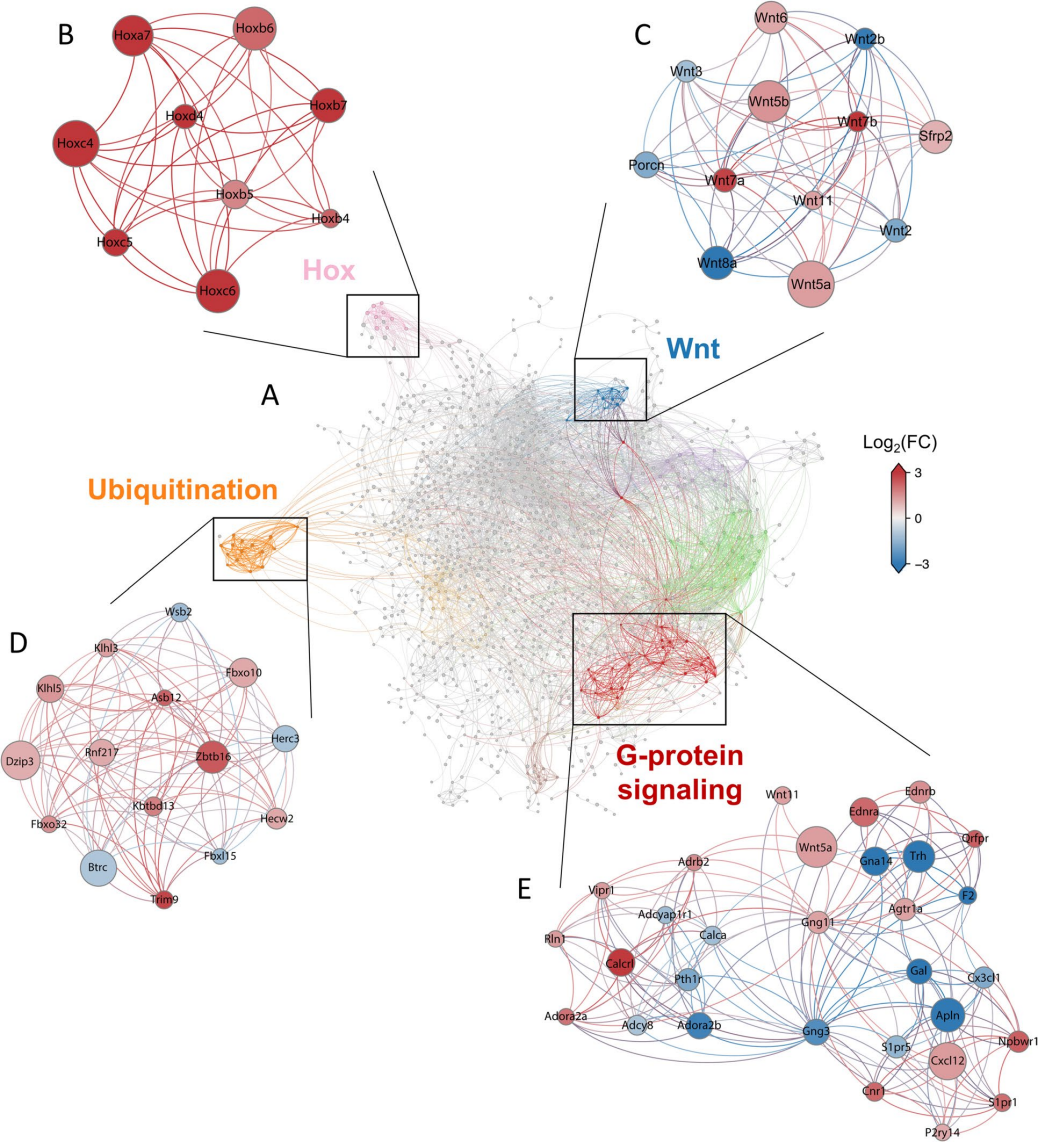
Interaction networks reveal changes in various functional modules. **A** Protein–protein interaction network based on the differentially expressed genes between E7.5 and E8.5. Colored clusters represent structurally coherent network modules identified using LowEnDe [40]. Purple cluster, Growth factors; Green cluster, Lipoprotein metabolism; Yellow cluster, Immune system. **B**-**D** Expanded versions of the Hox (**B**), Wnt (**C**), ubiquitination (**D**) and G-protein coupled receptor signaling (**E**) modules are shown to highlight the genes included in each. Nodes are colored by Log_2_(Fold Change), node size by Log_2_(CPM). Significantly upregulated genes at E8.5 are in red, downregulated genes at E8.5 are in blue

To get a closer image of the changes associated with the transition from head to trunk development, we built a protein–protein interaction (PPI) network (Fig. 2A and Additional file 3: Fig. S2) based on the differentially expressed genes between E7.5 and E8.5, as obtained from StringDB [37]. To focus our analysis on the most relevant interactions, we computed the metric backbone of this PPI network [38], which removed all redundant interactions and has been shown to help identifying genes and interactions responsible for core cellular programs [39]. Next, we identified structurally coherent network modules using LowEnDe [40], with an in-house developed algorithm based on spectral decomposition and information theory. Our interpretation is that these network modules may represent core development functions that are responsible for key aspects of the head to trunk transition.

One of the resulting clusters comprised the *Hox* genes (Fig. 2B) that we had already identified in our manual inspection of the differentially regulated genes, thus serving again as an internal validation of the approach. Another prominent cluster was associated with ubiquitination processes (Fig. 2D) enriched in genes encoding for E3 ligases, the key determinants of substrate specificity of the ubiquitin proteasome system [41]. This cluster contains a mix of up and downregulated genes, suggesting a switch in global ubiquitination patterns during the head to trunk transition that could impact general cellular functions by changing the availability of components involved in those processes. Particularly interesting in this module is Btrc, known to promote β-catenin ubiquitination and its subsequent degradation [42–44], which has been shown to also interact with components of several other signaling pathways and regulators of cell proliferation [45–47]. Indeed, the PPI network also identified several clusters composed of genes involved in different signaling pathways, indicating the existence of a substantial change in the signaling activities governing cell function when embryos engage in trunk formation.

One of those signaling-related clusters particularly prominent in the PPI network was composed of genes involved in G-protein coupled receptor signaling (Fig. 2E). This cluster included both up- and down-regulated genes and revealed a switch in the gamma subunits of the heterotrimeric G protein complexes, from Gng3 to Gng11, which could impact the selection of the pathways supported by the complex.

Those general changes in G protein-mediated signaling might play a role in the functional changes associated with Wnt signaling during the head to trunk transition. In particular, the PPI network showed connections between the G-protein cluster and *Wnt5a* and *Wnt11*. This connection might expose a regulatory switch, considering that these Wnt factors are known to signal through non-canonical pathways [48–50] and their activity is essential when the embryo enters trunk development [18, 19]. It will be therefore interesting to determine whether the changes observed in the molecular composition of the G-protein signaling cluster from E7.5 to E8.5, promotes activation of the non-canonical Wnt/Ca^2+^ pathway by Wnt5a and Wnt11 [50] when the embryo engages in axial extension. A more prominent involvement of the non-canonical Wnt signaling downstream of Wnt5a when entering trunk development was also suggested by the upregulated *Sfrp2* expression at E8.5 (Fig. 2C), since Sfrp2 redirects Wnt signals from Fz7 to Ror2, stabilizing the Wnt5a-Ror2 complexes that mediate Wnt5a activity during body axis development [51, 52]. The possible involvement of *Sfrp2* in this process is also supported by genetic data showing its requirement during trunk axial extension redundantly with *Sfrp1* [53].

Another of the relevant changes in Wnt signaling associated with the head to trunk transition is the switch from *Wnt*3 to *Wnt3a* functional dependency [17, 44]. In our datasets, *Wnt3* was downregulated at E8.5, fitting with its functional dynamics. *Wnt3a* expression levels, however, did not change from E7.5 to E8.5. This contrasts with the known *Wnt3a* functional requirements, as it is essential during trunk development but seems to be either inactive or functionally limited at earlier developmental stages given its inability to replace for *Wnt3* [44]. This could suggest that stimulation of Wnt3a functional activity during axial extension might result from expression changes in additional factors modulating Wnt signaling at different levels of the pathway. Consistent with this, a stabilizing Axin2 mutation impacting differently canonical Wnt signaling in the progenitor region of E7.5 and E8.5 embryos [54], suggests fundamental changes in Wnt signaling as embryos engage in axial extension. A prominent candidate to be involved in differential Wnt regulation is *Porcn*, which codes for a molecule that introduces a palmitoleoyl moiety into a highly conserved serine residue of the Wnt ligands [55, 56]. Given the essential role of *Porcn* during gastrulation [57], it was somewhat surprising to find a reduction of *Porcn* expression levels in the posterior epiblast at E8.5. This reduction was confirmed by direct measurement of *Porcn* transcript levels in the posterior epiblast of E7.5 and E8.5 embryos by RT-qPCR, which revealed a significant down-regulation of this gene from E7.5 to E8.5 (Additional file 1: Fig. S3A and Table S2), thus consistent with the RNA-seq data. In addition, analysis of the single cell RNA-seq data from Pijuan-Sala et al., 2019 [23] also showed that, while *Porcn* transcripts were readily observed in the caudal epiblast cluster of E7.5 embryos, they were absent from the same cluster at E8.5 (Additional file 1: Figs S3B and C). A reduction of *Porcn* expression levels when embryos enter the axial extension phase was also observed in the in situ expression patterns reported for this gene [57]. Whether this reduction plays a role in the Wnt signaling switch associated with the head to trunk transition is unclear. Intriguingly, it has been reported that pharmacological inhibition of Porcn impacted differently canonical and non-canonical Wnt signaling in a cell line assay [58], and Wnt3a was also shown to activate Wnt signaling in the absence of Porcn [59], suggesting that the Porcn-mediated modification might not be a universal requirement for Wnt signaling.

### Wnt signaling dependency on Porcn during axial extension

**Fig. 3:**
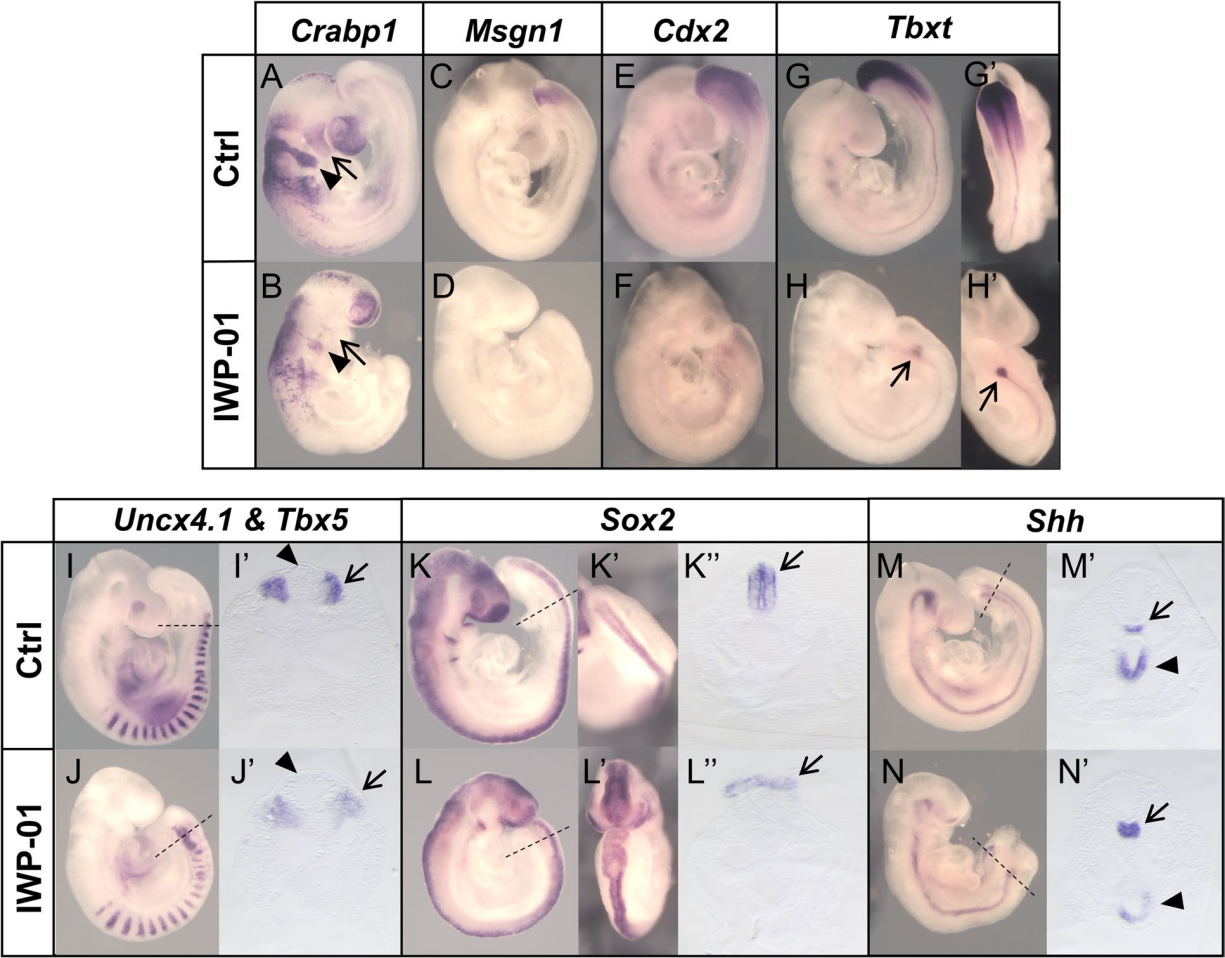
Impact of Porcn activity during axial elongation. Embryos were cultured for 24h (E8.5 to E9.5) in the presence or absence of the Porcn inhibitor, IWP-01. Whole-mount in situ hybridization with *Crabp1* (A-B)*, Msgn1* (C-D)*, Cdx2* (E–F), *Tbxt* (G-H’), *Uncx4.1 & Tbx5* (I-J)*, Sox2* (K-L) and *Shh* (M–N) probes. G’, H’, K’ and L’ show dorsal views of the posterior end of the respective embryos. I’, J’, K’’, L’’, M’ and N’ show transverse sections at the axial levels indicated by the dashed lines in the respective embryos. Arrows and arrowheads in A and B indicate first and second branchial arches, respectively. *Tbxt* expression in the caudal region is reduced to a small spot (arrows in H and H’). Arrows and arrowheads in I’ and J’ indicate paraxial mesoderm and neural tube, respectively. The neural tube (arrows in K’’ and L’’) becomes a flat structure in the posterior end of the IWP-01-treated embryo. Arrows and arrowheads in M’ and N’ emphasize *Shh* expression in the notochord and gut, respectively

We tested the effect of blocking Porcn activity on axial extension by incubating E8.5 embryos in vitro in the presence or absence of the Porcn inhibitor IWP-01. Our culture conditions allowed normal progression of development, with the embryos attaining typical E9.5 morphology within 24 h of incubation (Fig. 3). The presence of the inhibitor affected development in different ways. The brain structures were seriously reduced in size, likely affecting mainly the midbrain and anterior hindbrain structures, which also led to a substantial reduction in migratory cranial neural crest cells (Figs. 3A and B, arrows). These features are consistent with the inhibition of Wnt1 signaling [60], thus serving as an internal control for IWP-01 activity. IWP-01 treated embryos underwent considerable extension at the caudal embryonic end, although they eventually became truncated. *Uncx4.1* expression indicated the presence of paraxial mesoderm along the whole anterior posterior axis, presenting fairly normal-looking somites for a considerable extent of the trunk, but losing segmental patterns towards the end of the axis (Fig. 3I-J’). The *Uncx4.1* signal almost reached the caudal embryonic end, indicating that the presomitic mesoderm (PSM) was strongly reduced or absent, an idea also suggested by the lack of *Msgn1* signal (Fig. 3C and D). *Sox2* expression indicated that IWP-01-treated embryos also developed a spinal cord, morphologically normal at the axial levels containing identifiable somites and becoming a wider flattened structure in the region containing the disorganized *Uncx4.1* expression (Fig. 3J’, L’’). Importantly, even in the area showing abnormal neural and paraxial mesodermal patterns, IWP-01 treated embryos contained a single neural tube. The axial truncation in the context of a disorganized paraxial mesoderm and enlarged spinal cord could indicate an exhaustion of NMCs derived from accelerated progenitor differentiation at the expense of self-renewal. The lack of *Cdx2* expression at the caudal end of IWP-01-treated embryos is consistent with this hypothesis (Fig. 3E, F). Interestingly, *Shh* expression showed that the notochord also became truncated in the region where the paraxial mesoderm and the neural tube lose normal patterns (Fig. 3M-N’). *Tbxt* expression was reduced to a small spot beneath the neural tube (Fig. 3H, H’), roughly corresponding to the position of the caudal end of *Shh* expression, indicating that it could represent the posterior end of the notochord.

Our data indicate that during axial extension Wnt signaling involves a combination of Porcn dependent and independent activities. This contrasts with the essential role of Porcn during gastrulation [57]. Interestingly, IWP-01 seemed to have very limited effect on trunk extension. While inefficient IWP-01 activity cannot be formally ruled out (although it clearly affected other embryonic areas), this observation is consistent with the observed absence of *Porcn* transcripts in the caudal epiblast of E8.5 embryos, thus suggesting that Wnt activity in this embryonic region is mostly Porcn-independent. However, axial extension, as well as paraxial mesoderm and spinal cord development (among the most relevant sensors of Wnt activity) were clearly affected caudal to the axial level roughly corresponding to the transition into tail bud-dependent elongation, thus suggesting different requirements for the control of epiblast-driven and tail bud-dependent axial elongation. A change in Porcn dependence when the embryo switches to tail development fits with the measured *Porcn* transcript levels in the progenitor region of E8.5 and E9.5, because although not statistically significant, they were higher in the older embryos (Additional file 1: Fig. S3A). Even considering the abnormal features of the neural and paraxial mesoderm in the tail of IWP-01 treated embryos, their presence throughout the whole AP axis of these embryos, differs from the duplicated neural tubes replacing the paraxial mesoderm characteristic of the *Wnt3a* mutant embryos [61]. This indicates that during axial elongation Wnt3a signaling might include Porcn-independent activities, an effect previously observed in a cell culture context [59]. The malformations observed at the caudal end of the IWP-01-treated embryos suggest that the required equilibrium between differentiation and self-renewal of NMC cells in the tail bud might also entail proper balance of Porcn-dependent and Porcn independent Wnt activities.

### Chromatin accessibility landscape of the posterior epiblast in the developing embryo

**Fig. 4:**
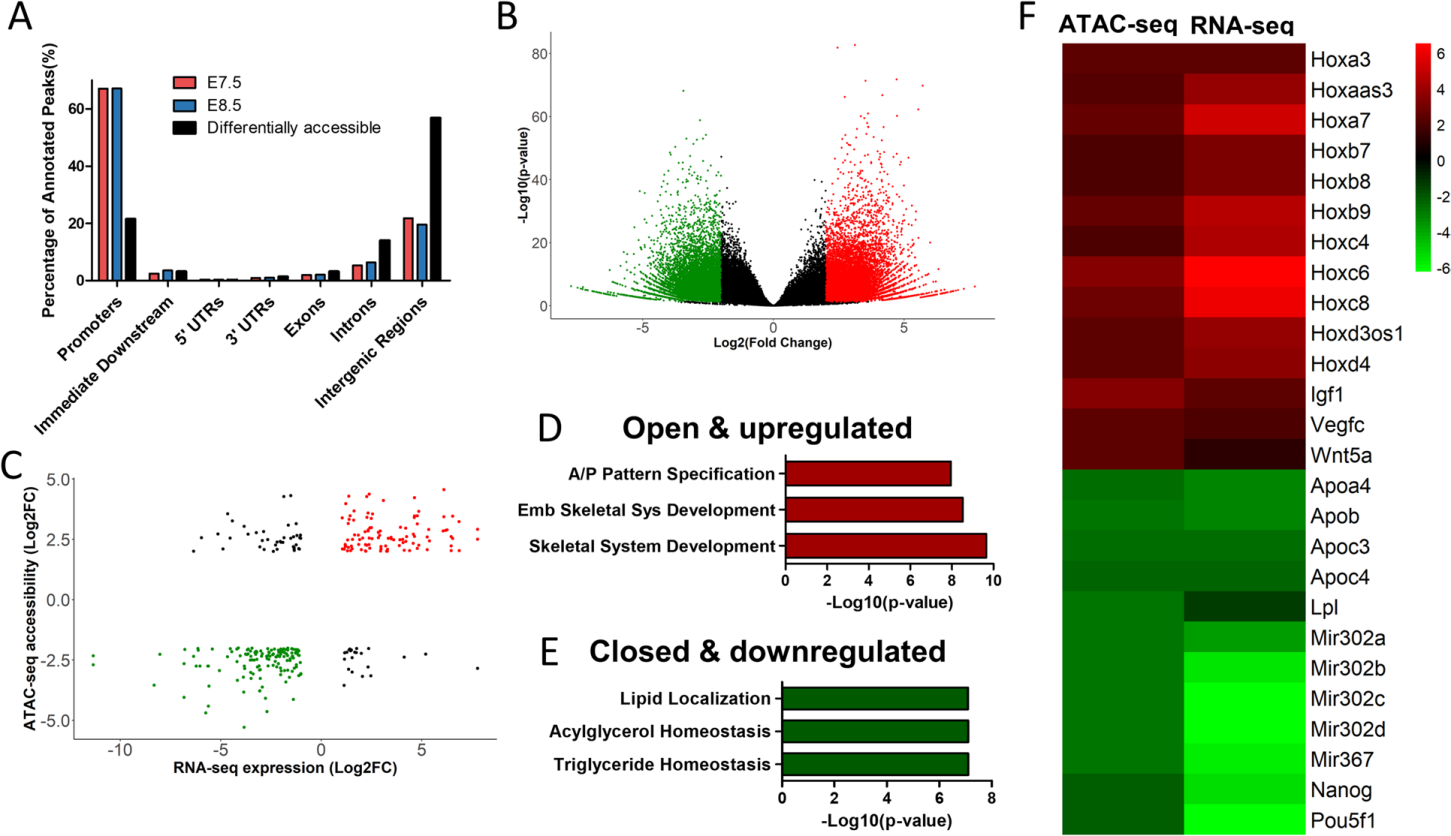
Integration of genome accessibility and gene expression data. **A** Genomic distribution of ATAC-seq peaks identified at E7.5 (red), E8.5 (blue) and distribution of only the differentially accessible peaks (black). **B** Volcano plot of ATAC-seq peaks (|Log_2_(Fold Change)|≥ 2 & *p*-value < 0.05). Significantly open regions at E8.5 in red, closed regions at E8.5 in green and non-significant in black. **C** Scatterplot showing correlation between genomic accessibility and gene expression. Significantly accessible and upregulated genes in red, closed and downregulated genes in green. **D** Top 3 GO biological process terms of positively regulated genes at E8.5 (red group in **C**), Abbreviations: Emb, Embryonic; Sys, System, A/P, Anterior/Posterior. **E** Top 3 GO biological process terms of negatively regulated genes at E8.5 (green group in **C**) (**F**) Heatmap of Log_2_(Fold Change) of ATAC-seq and RNA-seq signals

To understand the regulation behind the changes observed in gene expression, we mapped global chromatin accessibility profiles. For this, we performed the Assay for Transposase-Accessible Chromatin with sequencing (ATAC-seq) [62] from tissues of the same regions and timepoints as those used for RNA-seq. Principal component analysis separated the samples by timepoint (Additional file 1: Fig. S1B), indicating the presence of distinct chromatin accessibility profiles at these two developmental stages. Both E7.5 and E8.5 datasets had a similar chromosomal distribution of accessible regions, with two thirds mapping to promoters and about 20% to intergenic regions (Fig. 4A). Differential analysis of the two datasets identified 18,197 regions with increased chromatin accessibility (open regions), and 11,087 with decreased accessibility (closed regions) at E8.5 relative to E7.5 (Fig. 4B and Additional file 4: Table S3). Interestingly, the differentially accessible peaks followed a distribution different to that observed for the individual datasets, with most peaks (57%) mapping to intergenic regions, 14% to introns and the contribution of promoters being reduced to around 21% (Fig. 4A). This suggests that the transition between these developmental stages is to a large extent associated with a switch in regulatory elements. In addition, the finding that there are around ten times more genomic regions changing accessibility profiles than differentially expressed genes suggests a high complexity in the regulatory mechanisms controlling the transcriptional switch associated with the head to trunk transition.

From the regions showing differential accessibility, only 1418 could be associated with an annotated gene within 5 kb. Integrative analysis of transcriptomic and chromatin dynamics by crossmatching these 1418 regions with the differentially expressed genes (*n* = 3758) identified 300 genes in common, of which 238 showed consistent regulation at both chromatin and transcriptomic levels (Fig. 4C) (i.e., upregulated transcripts close to regions that became accessible or downregulated transcripts close to regions that lost accessibility). The remaining 62 regions might represent inhibitory elements. These observations indicate that only a very small proportion of the regions that change accessibility during the head to trunk transition are predicted to control the closest annotated transcriptional unit, thus further complicating the understanding of the regulatory processes controlling the head to trunk transition. Analysis of GO terms of this restricted group revealed an enrichment in anterior/posterior pattern specification and skeletal system development, in genes which are both accessible and upregulated at E8.5 (Fig. 4D). These include several *Hox* genes, most particularly those of central and posterior paralog groups (Fig. 4F), which might reflect the activation of enhancers within the *Hox* clusters upon sequential global opening of the clusters during axial extension [63]. The group of less accessible and downregulated genes include genes related to stem cell pluripotency and proliferation (Fig. 4F), like the already mentioned *Pou5f1* and *Nanog.* This is consistent with the known position of relevant regulatory regions for these genes [64, 65]. This group also included the *miR-302/367* cluster, important for stem cell maintenance and repression of cell differentiation [66].

GO terms of the less accessible and downregulated genes were enriched for triglyceride homeostasis and lipid metabolism (Fig. 4E), including several *Apo* genes as well as *Lpl*, that catalyzes the hydrolysis of triglycerides (Fig. 4F). These observations indicate that the head to trunk transition is associated with changes in lipid metabolism, which have the potential to impact the activity of various signaling pathways. For instance, lipid modifications have been shown to be essential to generate functionally competent Wnt and Hedgehog molecules [67, 68]. In the case of Wnt ligands, they contain several lipidic modifications, including the above-mentioned palmitoleoylation, which have been shown to affect differently the functional activity of different Wnt molecules [58, 69] and, as already discussed above, could be involved in the implementation of the functional switch in Wnt signaling associated with the head to trunk transition. Interestingly, in Drosophila embryos lipid-modified Hedgehog and Wingless require association with lipoproteins for long-range spreading of their activity [70], and Wnt5a has also been shown to associate with lipoprotein particles for long distance regulation of hindbrain development [71]. In our datasets, several genes encoding for lipoprotein components are downregulated at E8.5. While this could be related to changes in the transport of lipid nutrients to the developing embryo, as shown for Apob during mouse embryogenesis [72], it could also impact Wnt and Hedgehog activities by determining the spatial range of their activity at different developmental stages.

### Transcription factor binding activity in the posterior epiblast

**Fig. 5:**
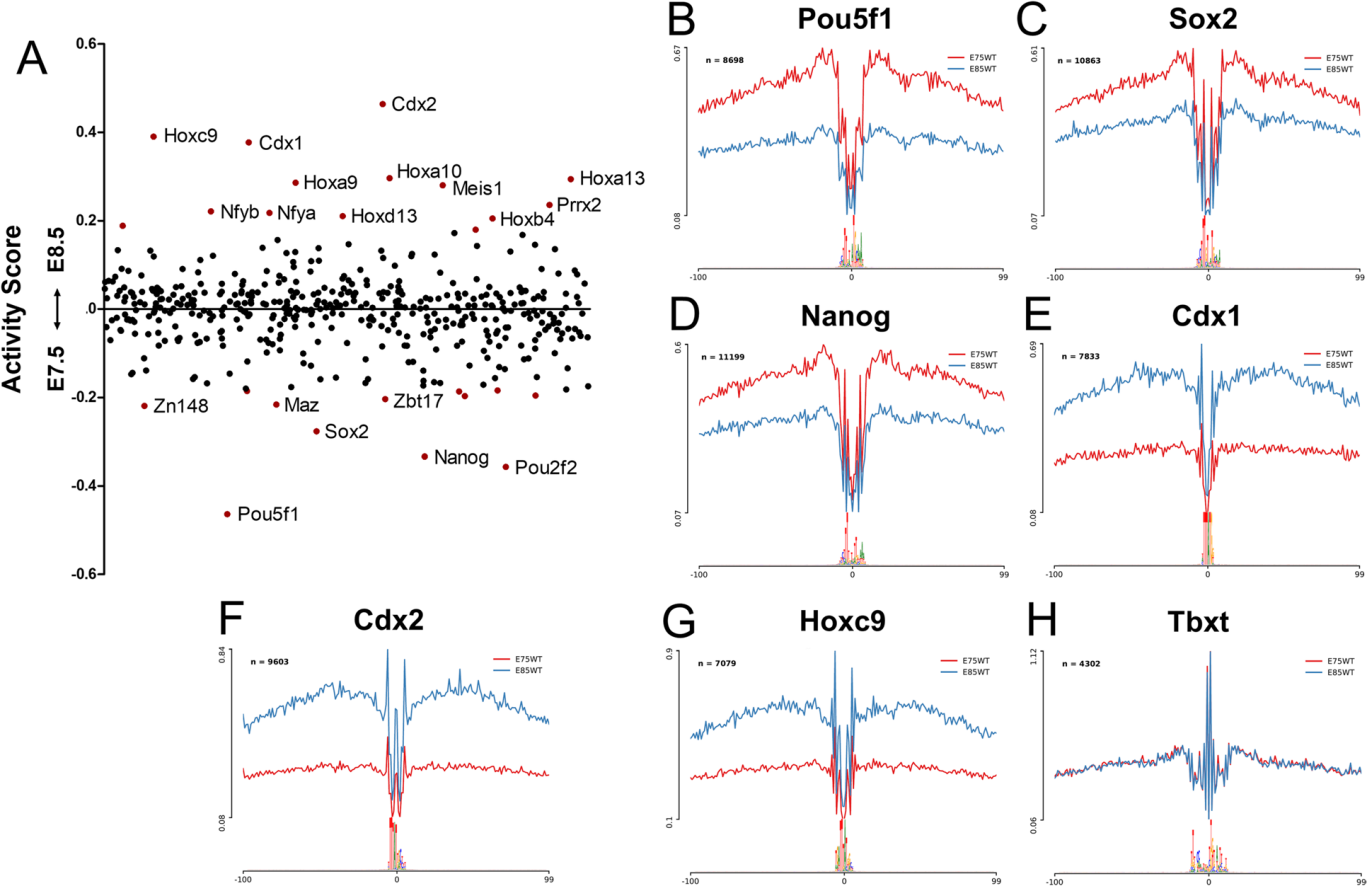
TF activity dynamics during the head to trunk transition. **A** Scatter plot of TF activity dynamics between E7.5 and E8.5. The y-axis represents the differences in TF binding activity. Each point represents a TF, points colored in red have significantly different activity scores (*p*-value < 0.05). Labelled points have a differential |Activity Score|> 0.2. (B-H) Average ATAC-seq profiles of Pou5f1 (**B**), Sox2 (**C**), Nanog (**D**), Cdx1 (**E**), Cdx2 (**F**), Hoxc9 (**G**) and Tbxt (**H**) binding sites. Red profiles correspond to E7.5, blue profiles to E8.5, n indicates the number of binding sites used to calculate the average profiles

To assess how the modification of the chromatin accessibility profiles between E7.5 and E8.5 was reflected in the binding profiles of TFs known to be involved in developmental processes, we searched for TF footprints in our ATAC-seq datasets using HINT-ATAC [73]. We found several TFs with a significant difference in activity score between the two developmental stages (Fig. 5A). At E7.5 we observed a higher activity score for TFs involved in pluripotency, like Pou5f1, Nanog and Sox2. The average ATAC-seq profiles around the binding sites of each of these TFs revealed that, although at a lower level and in a reduced number of regions, binding activity was still detected at E8.5 (Fig. 5B-D). This might reflect a change in the functional profile of those factors as development proceeds. For instance, while Sox2 and Pou5f1 are required for pluripotency [25, 74], later in development they are involved in trunk elongation (Pou5f1) or in neural tube development (Sox2) [75–77]. At E8.5 the highest activity scores were provided by Cdx2, Cdx1, and several posterior Hox proteins (Fig. 5A). Interestingly, their ATAC-seq profiles showed shallow footprints at E7.5 (Fig. 5E-G), revealing that binding of these factors to their genomic targets mostly starts when the embryo engages in trunk development. These observations fit the genetic data showing that in the absence of Cdx activity, mouse embryos are truncated at the head to trunk transition [14, 16, 78], thus indicating that the functional requirement for these genes starts at this transition. Conversely, the binding profile of Tbxt (Brachyury), another of the main regulators of axial extension [13], was similar at E7.5 and E8.5 (Fig. 5H). This might reflect the high overlap in the genomic binding profiles of Eomes and Tbxt despite their distinct and non-redundant functional requirement during gastrulation and axial extension, respectively [6, 15, 78, 79]. Together, these results highlight a change in the main regulatory networks involved in each of these developmental stages, which is reflected by the activity levels of specific TFs.

### Testing a potential enhancer region of *Wnt5a*

**Fig. 6:**
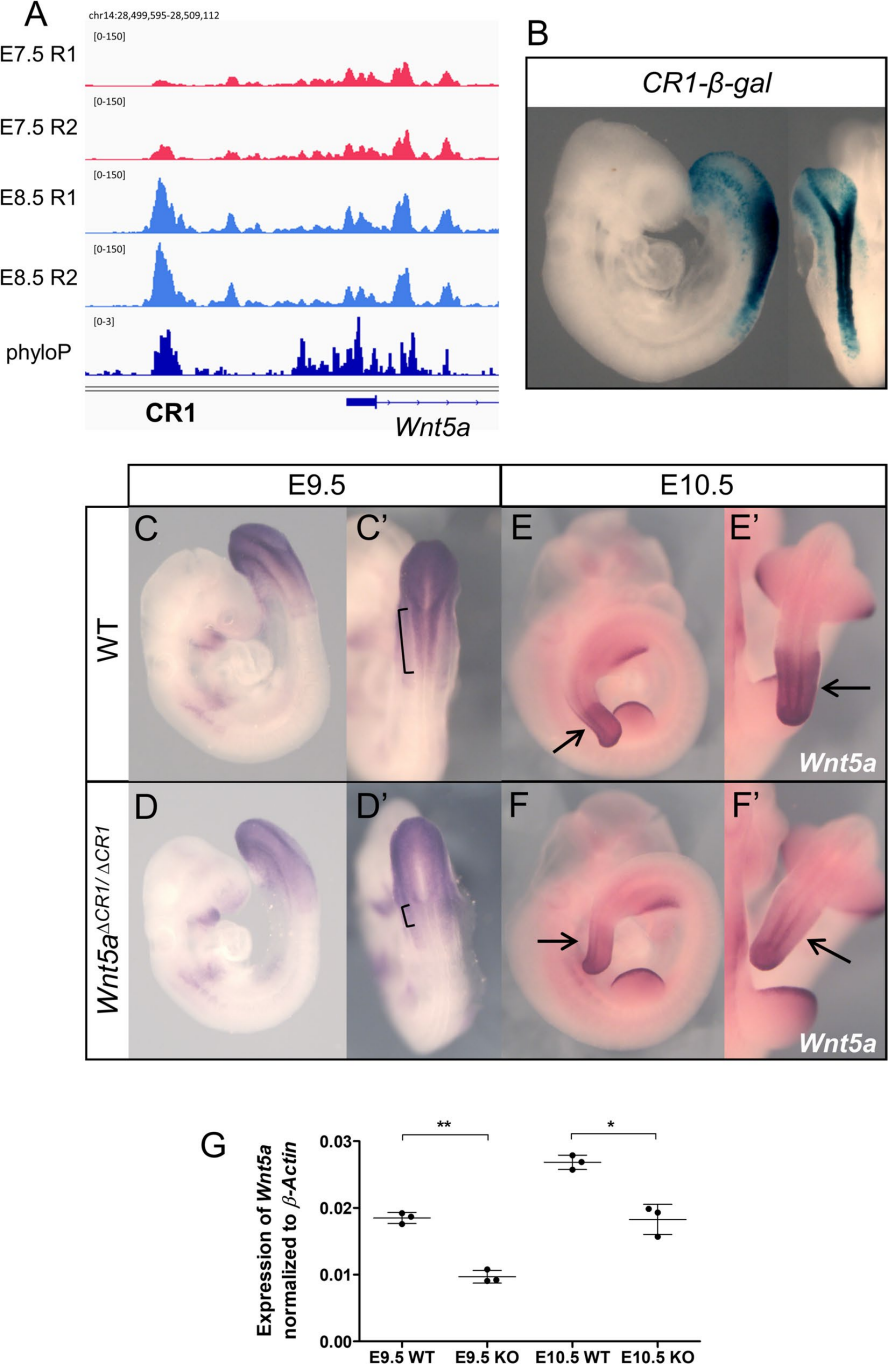
Characterization of Wnt5a enhancer, CR1. **A** ATAC-seq tracks showing accessibility profiles in the CR1 region*.* Phylogenetic conservation data (phyloP) [80] is shown in dark blue. **B** β-gal staining of *CR1-β-gal* transgenic embryo (*n* = 3/10), also showing dorsal view of the caudal region. **C**-**F** Whole-mount in situ hybridization of wild type and *Wnt5a*^*∆CR1/∆CR1*^ embryos at E9.5 and E10.5 using a probe for *Wnt5a*. (C’ and D’) Dorsal view of the posterior end of the embryo emphasizing the reduction of *Wnt5a* expression in the neural tube (brackets). At E10.5 *Wnt5a* expression is reduced in the PSM (arrows in E’ and F’). (G) RT-qPCR analysis of *Wnt5a* gene expression in wild type and *Wnt5a*^*∆CR1/∆*^.^*CR1*^ embryos at E9.5 and E10.5. *Wnt5a* expression is normalized to *β-Actin*. Three individual embryos were used for each condition. Values provided in Additional file 1: Table S4. Error bars indicate the standard deviation; **, *p*-value < 0.01 and *, *p*-value < 0.05

From the 238 ATAC-seq peaks associated with differentially regulated genes, we focused on a region approximately 3.3 kb upstream of *Wnt5a* transcriptional start site that becomes accessible at E8.5 (Fig. 6A). This region is highly phylogenetically conserved among mammalian species [80], thus making it a candidate to regulate *Wnt5a* expression when the embryo engages in trunk development. We will refer to this region as CR1.

We first tested the regulatory potential of this putative enhancer using a reporter assay in mouse embryos. Transgenic embryos consistently displayed reporter expression in the posterior epiblast and emerging neural tube, a pattern closely resembling *Wnt5a* expression (Fig. 6B). This pattern is consistent with CR1 involvement in *Wnt5a* activation in the progenitor region during the head to trunk transition.

To directly explore this hypothesis, we generated CR1 deletion mutants (*Wnt5a*^∆*CR1*^). Whole-mount in situ hybridization suggested a reduction in *Wnt5a* expression levels in the emerging neural tube of *Wnt5a*^*∆CR1/∆CR1*^ embryos at E9.5 (Fig. 6C-D’, brackets), and in the PSM at E10.5 (Fig. 6E-F’, arrows). This downregulation was confirmed by quantitative RT-PCR, at both stages (Fig. 6G and Additional file 1: Table S4). These results suggest that, while the CR1 element participates in the regulation of *Wnt5a* expression in vivo, this regulation should also involve the activity of additional redundant enhancers that confer robustness to *Wnt5a* expression, able to keep a baseline *Wnt5a* transcription in *Wnt5a*^∆*CR1/*∆*CR1*^ mutants, thus allowing their full embryonic development. Despite the observed downregulation of *Wnt5a*, *Wnt5a*^∆*CR1/*∆*CR1*^ mutants developed normally, generating adult animals with no obvious phenotypic defects. This contrasts with *Wnt5a*^*−/−*^ mutants, where loss of *Wnt5a* leads to perinatal lethality, with embryos showing an absence of tail and a shortening of the anterior–posterior axis [18].

### Impact of RA signaling on the transition from head to trunk development.

**Fig. 7:**
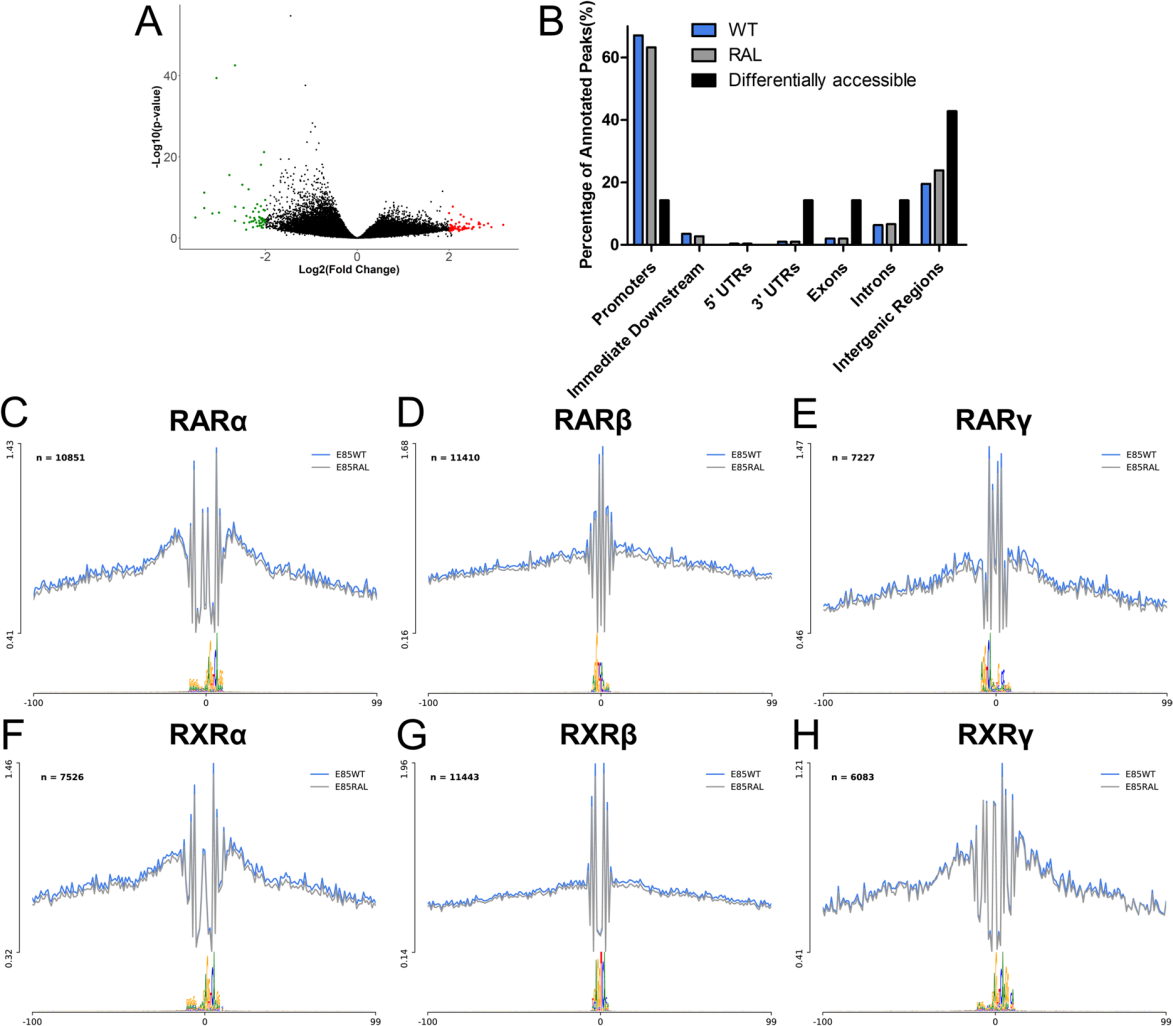
Impact of RA signaling in genome accessibility of the posterior epiblast. **A** Volcano plot of ATAC-seq peaks (*Raldh2*^*−/−*^ vs WT) (|Log_2_(Fold Change)|> 2 & *p*-value < 0.05). Significantly open regions in *Raldh2*^*−/−*^ are red, closed regions in *Raldh2*^*−/−*^ are green and non-significant in black. **B** Genomic distribution of ATAC-seq peaks identified in WT (blue), *Raldh2*^*−/−*^ (gray) and distribution of only the differentially accessible peaks (black). **C**-**H** Average ATAC-seq profiles of RARα (**C**), RARβ (D), RARγ (**E**), RXRα (**F**), RXRβ (**G**) and RXRγ (**H**) binding sites. Blue profiles correspond to wild type, gray profiles to *Raldh2*^*−/−*^, n indicates the number of binding sites used to calculate the average profiles

Genetic analyses revealed a fundamental role of RA signaling for proper transition from head to trunk development [21]. We therefore tested the extent to which this is associated with changes in chromatin accessibility. We generated a *Raldh2* mutant strain by introducing in frame stop codons in the second exon (Additional file 1: Fig. S4). Homozygous embryos for this strain showed a phenotype similar to that described for other previously described *Raldh2* mutants [21]. Comparison of the global accessibility profiles from the posterior epiblast region of E8.5 wild type and *Raldh2* mutant embryos revealed that only 120 peaks were differentially accessible between both conditions, including 54 regions with decreased and 66 regions with increased accessibility in the *Raldh2*^*−/−*^ mutants (Fig. 7A and Additional file 5: Table S5). Again, we observed that the differentially accessible peaks mapped mainly to intergenic regions (43%) (Fig. 7B), indicating that they might represent regulatory elements.

TF footprinting analyses showed no significant change in retinoic acid- and retinoid X receptor (RAR and RXR) binding activity in *Raldh2*^*−/−*^ mutants (Fig. 7C-H). This fits the notion that RA receptors are normally bound to retinoic acid response elements (RAREs) but kept inactive until bound by RA, eventually leading to recruitment of histone acetyltransferases and transcriptional activation [81]. Interestingly, only 12 of the regions that became differentially accessible contain RA receptor binding sites. Together, these observations suggest that RA activity at this developmental stage does not involve major changes in the genomic regions bound by RA receptors and that most of the differences in chromatin accessibility observed in the *Raldh2* mutants are not mediated by direct RA activity, most likely representing instead downstream effects of factors under direct RA regulation. The potential involvement of genes regulated by the 12 elements containing binding sequences for RA receptors in this or other RA-dependent regulatory processes will require direct experimental analyses.

### Evaluating potential *Nr2f2* enhancers for RA-mediated *Nr2f2* activation

**Fig. 8:**
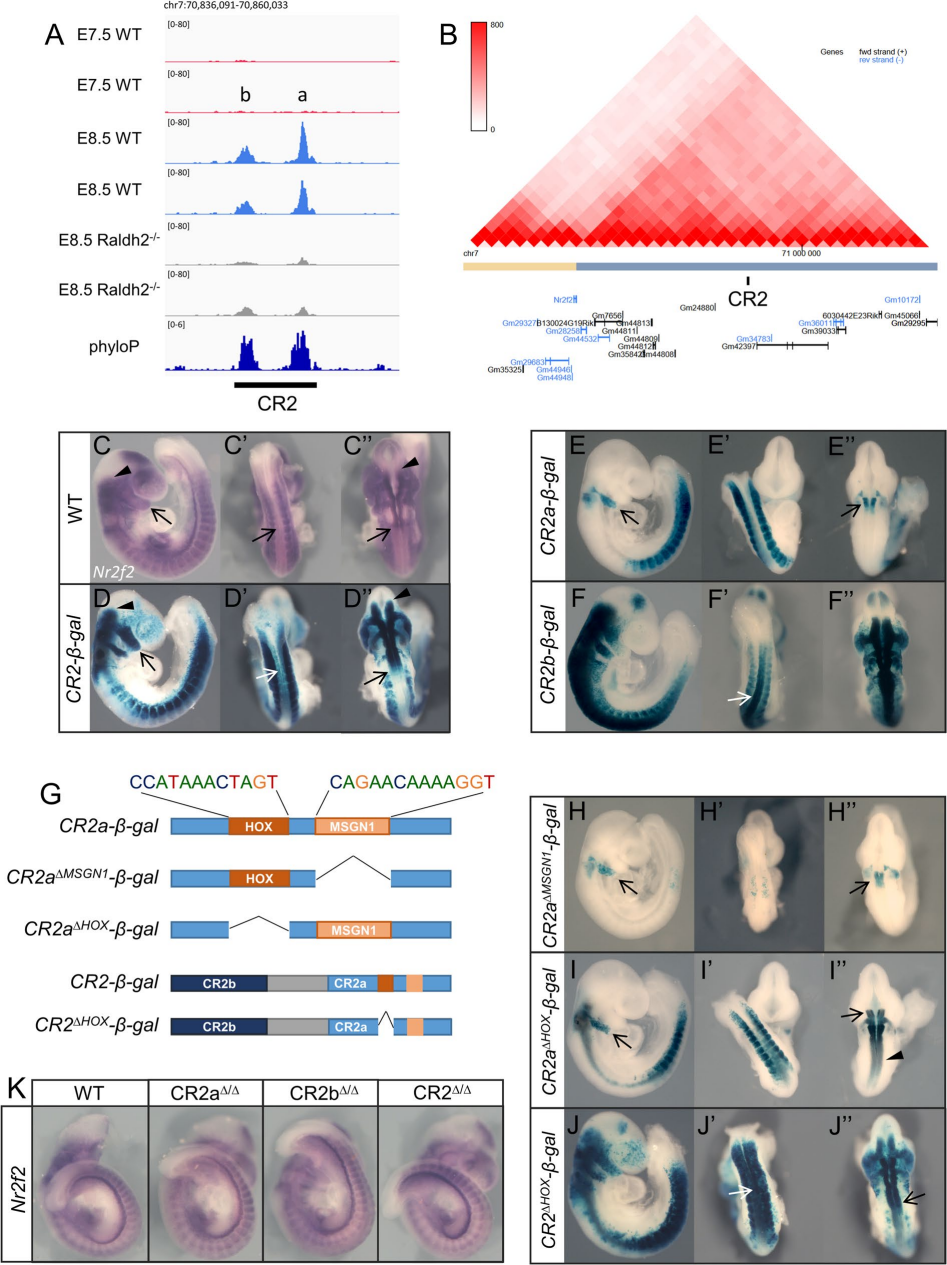
Characterization of the CR2 region. (A) ATAC-seq tracks showing accessibility profiles in the CR2 region*.* CR2 includes two peaks, *a* and *b*. Phylogenetic conservation data (phyloP) [80] is shown in dark blue. (B) Hi-C data from 3D genome browser [83] highlighting CR2 location in the same TAD as *Nr2f2*. (C-D’’) Comparison of *Nr2f2* expression pattern in wild type embryos by in situ hybridization (C–C’’) with *β-gal* reporter expression in *CR2-β-gal* transgenic embryos (*n* = 4/5) (D-D’’). Expression is present in the hindbrain (arrows in C’’ and D’’) but absent from the spinal cord (arrows in C’ and D’). (E-E’’) *β-gal* reporter expression in *CR2a-β-gal* transgenic embryos (*n* = 3/6). Reporter expression is restricted to the second branchial arch (arrow in E), rhombomere 5 (arrow in E’’) and somites from the forelimb level. (F-F’’) *β-gal* reporter expression in *CR2b-β-gal* transgenic embryos is extended to the anterior somites and neural tube (*n* = 3/3) (arrow in F’). (G) schematic representation of generated transgenic reporters for CR2a and CR2 regions lacking the specified TF binding sites. (H–H’’) *β-gal* reporter expression in *CR2a*^*∆MSGN1*^*-β-gal* transgenic embryos is limited to the second branchial arch (arrow in H), rhombomere 5 (arrow in H’’) (*n* = 3/3). (I-I’’) *β-gal* reporter expression in *CR2a*^*∆HOX*^*-β-gal* transgenic embryos expands up to rhombomere 3 (arrow in I’’) and along the neural tube (arrowhead in I’’) (*n* = 6/7). (J-J’’) *β-gal* reporter expression in *CR2*^*∆HOX*^*-β-gal* transgenic embryos is extended into the neural tube (arrows) (*n* = 3/5). (K) Whole-mount in situ hybridization of wild type, *CR2a*^*∆/∆*^, *CR2b*^*∆/∆*^ and *CR2*^*∆/*^.^*∆*^ at E9.5 using a probe for *Nr2f2*

From the regions that gained accessibility at E8.5 in a RA-dependent fashion we focused on a phylogenetically conserved region (we will refer to it as CR2) located within the same topologically associated domain (TAD) as *Nr2f2* (Fig. 8A, B), a gene that has been shown to be under the control of RA signaling [82]. We selected CR2 for further analysis because when tested in transgenic reporter assays it reproduced to a large extent the *Nr2f2* expression pattern (Fig. 8C-D’’), thus suggesting that it might be involved in the RA-dependent activation of *Nr2f2* expression. CR2 contains two distinct elements (CR2a and CR2b) (Fig. 8A). Both elements also gave well-defined activity profiles when tested individually in transgenic reporter assays. *CR2a-β-gal* embryos displayed staining in the somites starting from the forelimb level, in rhombomere 5 and in the second branchial arch neural crest (Fig. 8E-E’’). CR2b gave a much broader range of expression in the neural tube, including the whole hindbrain and the spinal cord, and in the neural crest migrating from the hindbrain into the branchial arches (Fig. 8F-F’’). It also activated expression in the most anterior somites, where CR2a activity was not observed. Together, these staining patterns indicate that CR2 activity in the somites, branchial arches, and hindbrain might result from the combined CR2a and CR2b activities. However, the strong *CR2b-β-gal* reporter staining in the spinal cord (Fig. 8F’, arrow) contrasts with the absence of staining in the same region of CR2 reporter transgenics (Fig. 8D’, arrow), suggesting that CR2a could block CR2b activity in this region.

To further analyze the mechanisms regulating CR2 enhancer activity and the interactions between CR2a and CR2b, we searched for the presence of TF binding sites within these elements with HINT-ATAC. We identified two TF footprints in CR2a, matching MSGN1 and HOX binding sites (Fig. 8G). Given the important role of these TFs in embryonic development, we assessed their contribution to CR2a enhancer activity by generating transgenic reporters for the CR2a element lacking each of these features. Transgenic embryos generated with CR2a lacking the MSGN1 binding site (*CR2a*^*∆MSGN1*^) lost almost completely reporter gene expression in the somites (Fig. 8H-H’’), consistent with the known role of *Msgn1* as a regulator of paraxial mesoderm [84, 85]. Conversely, transgenic embryos of CR2a reporters lacking the HOX binding site (*CR2a*^*∆HOX*^) did not affect somite expression, displaying instead extended reporter activity in the neural tube, including rhombomeres 3, 4 and 6 and the anterior spinal cord (Fig. 8I-I’’). This suggests a repressor rather than an activator role for Hox proteins in this enhancer, most particularly in the neural tube. We therefore tested whether the HOX binding site could also be involved in keeping CR2 inactive in the spinal cord by silencing CR2b activity in this embryonic region. Deletion of the HOX binding site from the CR2 reporter construct (*CR2*^*∆HOX*^) resulted in a substantial activation of reporter activity in the neural tube (Fig. 8J-J’’), although not as extensive as the pattern obtained with CR2b, indicating that it could indeed be part of the interaction mechanism between CR2a and CR2b.

We also identified binding sites for SMAD1 and SP5 in the CR2b element. Deletion of both sites resulted in the loss of reporter expression in most of the embryo, with some residual expression being detected in the hindbrain, neural crest and anterior spinal cord up until the trunk level (Additional file 1: Fig. S5). Hence, the CR2a and CR2b elements are regulated by distinct sets of TFs, further allowing these regions to drive robust gene expression patterns despite possible fluctuations in upstream TF levels [86].

Together, the reporter assays indicate the existence of regulatory interactions between the CR2a and CR2b elements to achieve a pattern of activity resembling *Nr2f2* expression. CR2 thus represents a case in which enhancer interactions, both positive and negative, play an important role in fine tuning gene expression, contributing to the production of sharp boundaries in the expression domains, similarly to what has been previously reported for other systems [87–89]. Our results also suggest that the RA-dependent opening of CR2a and CR2b might expose these elements to become activated by factors involved in the development of trunk and hindbrain structures.

To directly assess CR2 function and its potential relevance for *Nr2f2* expression, we generated mouse strains containing deletions of CR2a, CR2b and CR2*.* Homozygous mutant animals for each of these strains developed to term and the adults had no obvious phenotypic alterations, already indicating that these mutants kept *Nr2f2* expression, at least to a level allowing normal development. We confirmed this by whole-mount in situ hybridization showing that the *Nr2f2* expression pattern in homozygous mutant embryos for any of the deleted CR2 regions were similar to that observed in wild type embryos (Fig. 8K). This indicates that if CR2a and CR2b are indeed involved in *Nr2f2* expression as suggested by the reporter assays, other redundant enhancers might be present that ensure *Nr2f2* expression and prevent developmental arrest caused by the inactivation of this gene. A possible candidate for an enhancer able to maintain *Nr2f2* transcription in the absence of CR2 is the previously identified RAR-binding element (RARE) in intron 1 of *Nr2f2* [82]. However, further studies will be required to validate the role of this RARE in the regulation of *Nr2f2* and whether it interacts functionally with CR2.

## Conclusions

Overall, this study provides a comprehensive analysis to gain insights into the mechanisms regulating the remarkable changes in tissue activity associated with the transition from head to trunk development, combining differential screening with bioinformatic treatment of the resulting data. The specific treatment of the DEG profiles with the identification of modules in the PPI network revealed changes in Wnt signaling, ubiquitination and the basic machinery of G-protein-mediated signal transduction that could engage in interactions resulting in a global functional output. In addition, our datasets can be used as a resource for future research not only to explore the role of other enhancer regions but also to delve into the mechanisms of gene regulatory networks involved in the head to trunk transition by combining it with gene knockout studies.

The high disparity in the number of changes in chromosomal accessibility and differentially expressed genes at the two developmental stages indicates that the control of the changes in gene expression might also be very complex. Consistent with this, the lack of obvious phenotypes upon deletion of regulatory elements that largely reproduce the expression profiles of the genes they might regulate, argues for the existence of a considerable degree of redundancies among regulatory mechanisms. This redundancy, which has been previously observed for other regulatory regions [90, 91], might confer robustness to developmental processes by providing protection against genetic and environmental perturbations [90, 92–94]. Future studies testing the effects of CR1 or any of the CR2 deletions in a different genetic background or combined with a heterozygous inactivation of the *Wnt5a* or *Nr2f2* genes, respectively, or with other potential regulatory elements may reveal phenotypic traits normally suppressed by functional redundancy among enhancers.

## Methods

### Mice and embryos

The embryos analyzed in this work were recovered from pregnant females at different developmental stages. For this, matings between mice with the relevant genotypes were set up overnight and the day of the vaginal plug was considered embryonic stage E0.5. To collect embryos, pregnant females were euthanatized by cervical dislocation, embryos recovered from the uteri by cesarean section and processed accordingly for the distinct analysis described below.

*Raldh2* mutants, CR1, CR2, CR2a and CR2b deletion mice were generated by CRISPR/Cas9 [95] on the FVB/J background. *Raldh2* mutant mice were generated by introducing in frame stop codons into the second exon of the gene (Additional file 1: Fig. S4). A sgRNA targeting the sequence AATGGCAGAACTCAGAGAGT was generated by in vitro transcription. Briefly, oligonucleotides Raldh2-gRNA-up and Raldh2-gRNA-down (Table 1) were annealed and cloned into the BssI sites of plasmid pgRNA-basic [96]. The sgRNA was transcribed from the resulting plasmid with the MEGAshortscript T7 Kit (Life Technologies) and purified with the MEGAclear Kit (Life Technologies). Cas9 mRNA was produced by in vitro transcription from the pT7-Cas9 plasmid [96] using the mMESSAGE mMACHINE T7 Ultra Kit (Life Technologies) and purified with the MEGAclear Kit (Life Technologies). The replacement ssDNA oligonucleotide containing three in frame stop codons followed by an EcoRI site (Raldh2-3X-Stop) (Table 1) was purchased from IDT. A mixture of 10 ng/ µl of Cas9 mRNA, 10 ng/µl of the gRNA and 10 ng/µl of the Raldh2-3X-Stop oligonucleotide was injected into the pronuclei of fertilized oocytes of the FVB/J background, using standard procedures [97]. The mutant allele was detected by PCR using primers Raldh2-F and Raldh2-MUT-R (Table 2). Targeting was confirmed by direct sequencing. Deletion mutants for CR1, CR2, CR2a and CR2b were generated as previously described [98], using two gRNAs targeting the border of the sequence to be deleted and one ssDNA oligo bridging the two sides of the deletion (Table 1) to increase the edition efficiency. In these cases, each gRNA was generated by annealing the relevant Alt-R®-CRISPR-Cas9 crRNA (targeting sequences in Table 1) with the Alt-R®-CRISPR-Cas9 tracrRNA (all purchased from IDT). 1µM of each gRNA was incubated with 100 ng/µl of theCas9 protein and 10 ng/µl of the replacement DNA and microinjected into the pronucleus of fertilized mouse oocytes. Identification of deletion mutants was performed by PCR using the oligonucleotides specified in Table 2. Positive founders were crossed with wild type mice to generate F1 heterozygous mice that were then used to build the mutant lines. Homozygous mutants were then generated by heterozygous crosses. Mice and embryos were genotyped by PCR using primers specified on Table 2.

**Table 1:** gRNA and ssDNA used for CRISPR/Cas9

Raldh2	Raldh-gRNA-up	AGGGAATGGCAGAACTCAGAGAGT
	Raldh-gRNA-down	AAACACTCTCTGAGTTCTGCCATT
	Raldh2-3X-Stop	CATATCCCATTTTCTTGTGTCCCTTCTGTAGATCTTTATTAACAATGA ATGGCAGAACTCAGAGTGATAGAATTCAGTGGGAGAGTGTTCCCTG TCTGTAATCCAGCCACAGGAGAGCAAGTGTGTGAAGTTCAAGAAG
CR1	gRNA_CR1_1	CCAGTGGCAGTATTCTGTGA
	gRNA_CR1_2	CTGTGTAGCCGTAGTTTGCC
	ssDNA	CCCCCTAACCTCAAGGGAGCCTTTGTCCCCCACAGGCTAGTGG CCAGTGGCAGTATTCTGGCCAGGAGGTGAGGGACTTCCACAA ACTGGAGGCTCTCCTTTGGGAGTCTTCCCCAGTGG
CR2	gRNA_CR2_1	TCTTTCGGTCGTTCCCAGAG
	gRNA_CR2_2	GATACAACCGTCTTCTAGCT
	ssDNA	CCATCGGGGCGGGTGAGACCTCTCAGCACACCCTCTGTCCCCT TCTTTCGGTCGTTCCCAGCTAGGGAACCAGGGCAAAGTTGGC CTGGGTGGGATGGTTCTAAGGGTGCAGGGTGAACA
CR2a	gRNA_CR2a_1	ATTGGAGGTGCACTGGGTGA
	gRNA_CR2_2	GATACAACCGTCTTCTAGCT
	ssDNA	GGATGCTGGTGTGTATGCTTGTATGTGCCTTTGGAGTCAGGGT ATTGGAGGTGCACTGGGGCTAGGGAACCAGGGCAAAGTTGG CCTGGGTGGGATGGTTCTAAGGGTGCAGGGTGAACA
CR2b	gRNA_CR2_1	TCTTTCGGTCGTTCCCAGAG
	gRNA_CR2b_2	TCTCCTGGGCATTATCTGCC
	ssDNA	CCATCGGGGCGGGTGAGACCTCTCAGCACACCCTCTGTCCCCT TCTTTCGGTCGTTCCCAGCCAGGTTCACCCCATTTCTTTTTATAA TCTTACTACATATTTAAAGGAGTCCCTTGCCT

**Table 2:** Primers used for genotyping

Raldh2	KO	Fw	GTTTTCTGATCTCCCAGATCTC
		Rv	TCTCCCACTGAATTCTATCAC
	WT	Fw	GTTTTCTGATCTCCCAGATCTC
		Rv	AACACTCTCCCACTCTCTGAG
CR1	KO	Fw	GTCTCTTCCATGAGTGCTGAG
		Rv	CTGCATTCTAAGAAGCAGTCC
	WT	Fw	ACCCACTTTCTACAGCAGATC
		Rv	CTGCATTCTAAGAAGCAGTCC
CR2	KO	Fw	GAGCCACACTGATTTCAGAGG
		Rv	TCATCCATACCCTCCAGCTAC
	WT	Fw	GAGCCACACTGATTTCAGAGG
		Rv	AGACGTTACAGTAACGTGCTC
CR2a	KO	Fw	TGAATTGACGTGAGAGGAAGG
		Rv	TCATCCATACCCTCCAGCTAC
	WT	Fw	TGAATTGACGTGAGAGGAAGG
		Rv	GGCTGATGTGAAGCATTGCAG
CR2b	KO	Fw	GAGCCACACTGATTTCAGAGG
		Rv	TTATCACAGACTGTGACCAAC
	WT	Fw	GAGCCACACTGATTTCAGAGG
		Rv	AGACGTTACAGTAACGTGCTC

### RNA-sequencing analysis

Posterior epiblasts of wild type mouse embryos at E7.5 and E8.5 were dissected and snap frozen. Total RNA was isolated from pooled samples with TRI Reagent following the manufacturer’s protocol. RNA samples were then resuspended in RNase-free water. RNA concentration and purity were determined on an AATI Fragment Analyzer (Agilent). RNA-seq from E7.5 and E8.5 tissues was performed using two separate biological replicates. Libraries were prepared from total RNA using the SMART-Seq2 protocol [99]. Sequencing was performed on Illumina NextSeq500, generating > 25 M single-end 75 base reads per sample. Reads were aligned to the reference mouse genome (mm10) using STAR [100]. Read count normalization and differential expression between samples was analyzed using DESeq2 [101]. RNA-seq data is available in the GEO accession database under the accession number GSE220246. K-means clustering was performed on the 1000 most variable genes using the standard R function ‘kmeans()’. The elbow method was used to determine the number of clusters to use for this analysis. Gene ontology enrichment analysis was performed using PANTHER [102, 103], by selecting for biological processes using Fisher’s Exact test and False Discovery Rate. No background gene list was used. Gene ontology results presented are ranked by Fold Enrichment.

To assemble the PPI network, the DEG were filtered according to the following criteria: log of count per million (logCPM) > 1; absolute log fold-change (logFC) > 1; and false discovery rate (FDR) < 0.05. All possible interactions between DEGs were retrieved from the STRING v11 protein–protein interactions database [37]. The mouse transcriptome network was then constructed from the set of expressed genes and their corresponding STRING PPI. We casted this network as a weighted graph, where edge weights (given by the STRING PPI scores) denote the probability of the connected genes interacting and thus jointly contributing to a specific function. To remove redundant edges and focus our attention on the most important interactions we extracted the (metric) backbone of the mouse transcriptome network [38]. The metric backbone is a subgraph that is sufficient to compute all shortest paths in the network, thus removing edges that break the triangle unequally (and are therefore redundant regarding the shortest paths). This network retains all metric edges and preserves all the nodes in the original network [38, 104]. We have previously used the metric backbone of transcriptome networks to identify biologically relevant genes and their interactions [39]. Network modules, i.e., structurally coherent structures in the transcriptome network backbone were identified using LowEnDe [40], an in-house developed algorithm based on the spectral decomposition of the adjacency matrix coupled with information theory to identify overlapping modules in weighted graphs. Importantly, in this method genes may participate in more than one module at the same time, reflecting the possible participation of genes in multiple cell functions.

### Embryo culture with Porcn inhibitor

Wild type E8.5 embryos were dissected, in cold GMEM (Sigma #G5154), keeping the yolk sac intact. Embryos were cultured in 60% Rat serum, 40% GMEM and Pen/Strep (Gibco #15,070,063). For embryos cultured with Porcn inhibitor, 500 nM of IWP-01 (MedChem express #HY-100853) was added as in [58], whereas for control embryos an equal volume of DMSO was added. Embryos were cultured for 24 h in a rotator bottle culture apparatus (B.T.C. Engineering, Milton, Cambridge, UK) at 37˚C, 65% O_2_. Three embryos were cultured per tube in 1.5 ml of media. Embryos were collected after 24 h, dissected and fixed in 4% PFA at 4˚C overnight. They were then processed for in situ hybridization, 2 embryos were stained per probe and condition, showing similar patterns. In addition, the structure of the neural tube was also assessed in the sections of embryos stained for other markers, showing highly reproducible patterns.

### ATAC-seq

Posterior epiblasts of mouse embryos were collected to 500 µl of cold M2 (Sigma #M7167), spun down to remove supernatant and incubated with 500 µl of Accutase (Sigma #A6964) for 30 min at 37ºC, with shaking at 600 rpm, to dissociate the tissue into single cells. ATAC-seq was performed as previously described [62], using two separate biological replicates for each condition. The amplified libraries were double-step size selected (0.5 × followed by 1x) using SPRIselect (Beckman Coulter #B23317) according to manufacturer’s instructions. Pooled ATAC-seq libraries were sequenced on a NextSeq500 (Illumina) at 50 M paired-end 75 base reads per sample.

### ATAC-seq data analysis

Fastq files were processed with GUAVA v1, following the recommended guidelines [105]. GUAVA enables pre-processing of raw sequencing reads, mapping of reads to a reference genome, peak calling and annotation, as well as differential analysis between samples. ATAC-seq data is available in the GEO accession database under accession number GSE220245. All genome browser tracks were captured using Integrative Genomics Viewer [106]. Phylogenetic conservation data for multiple alignments of 59 vertebrate genomes to the mouse genome (mm10.60way.phyloP60way) was obtained from phyloP directory [80] and loaded into IGV. Hi-C data was obtained from 3D genome browser [83] using the ‘mm10 mESC Bonev_2017-raw’ dataset [107] at 40 kb resolution. To visualize our candidate regions within the context of this dataset we loaded a BED file with the coordinates of our candidate regions to UCSC Genome Browser [108] and loaded this session into the 3D genome browser.

ATAC-seq data was analyzed for TF footprints using HINT [73]. Replicates were merged to increase read depth and processed with “rgt-hint footprinting” command. Footprint motifs were matched to HOCOMOCO database [109] with “rgt-motifanalysis matching” and then further assessed for differential motif occupancy with the “rgt-hint differential” command.

### β-Galactosidase transgenics

For reporter analyses, candidate regions identified by ATAC-seq data were amplified by PCR from mouse genomic DNA (primers provided below, Table 3) and cloned upstream of a cassette containing the adenovirus 2 minimal late promoter, the β-galactosidase cDNA, and the polyadenylation signal from SV40 [110]. Transgenic mice were produced by pronuclear injection [97]. The β-galactosidase staining was performed as previously described [110].

**Table 3:** Primers used to amplify candidate regions for β-Galactosidase assays

CR1	F_CR1	TACTCGAGCTGCTGCTCTTGACTCTGAAG
	R_CR1	ATCTGCAGATGCTCTGGACTCCGAGGAAC
CR2	F_CR2	GACTCGAGGTGTCAGACCTGTGTAAATGC
	R_CR2	ACCTGCAGGGAGGAAATGTTGTTGTTTGG
CR2a	F_CR2a	TTACTCGAGGCTGTCTACAGTGACTCTGTG
	R_CR2	ACCTGCAGGGAGGAAATGTTGTTGTTTGG
CR2b	F_CR2	GACTCGAGGTGTCAGACCTGTGTAAATGC
	R_CR2b	ATCTGCAGGTAAGGAGCAGACTTCACGTC
CR2a^∆MSGN1^	F_ CR2a	TTACTCGAGGCTGTCTACAGTGACTCTGTG
	R_CR2a∆MSGN1	GCCGAATTCTACTAGTTTATGGGGCTGATG
	F_CR2a∆MSGN1	CGCGAATTCGACACTTGAAAGTACCAGTTC
	R_CR2	ACCTGCAGGGAGGAAATGTTGTTGTTTGG
CR2a^∆HOX^	F_ CR2a	TTACTCGAGGCTGTCTACAGTGACTCTGTG
	R_CR2a∆HOX	GCAGAATTCGGCTGATGTGAAGCATTGCAG
	F_CR2a∆HOX	GCCGAATTCAGTCAGAACAAAAGGTCTGAC
	R_CR2	ACCTGCAGGGAGGAAATGTTGTTGTTTGG
CR2^∆HOX^	F_CR2	GACTCGAGGTGTCAGACCTGTGTAAATGC
	R_CR2a∆HOX	GCAGAATTCGGCTGATGTGAAGCATTGCAG
	F_CR2a∆HOX	GCCGAATTCAGTCAGAACAAAAGGTCTGAC
	R_CR2	ACCTGCAGGGAGGAAATGTTGTTGTTTGG
CR2b^∆SMAD+SP5^	F_CR2	GACTCGAGGTGTCAGACCTGTGTAAATGC
	R_ CR2b∆SMAD + SP5	ATTCTAGAGTGGCTTCTGCTCCAGAGCTC
	F_ CR2b∆SMAD + SP5	CGTCTAGATTCTAAGAGACTCAGTGGCTC
	R_CR2b	ATCTGCAGGTAAGGAGCAGACTTCACGTC

### Whole-mount in situ hybridization

Whole-mount in situ hybridization was performed as previously described [111] using digoxigenin-labeled RNA antisense probes. For the genetically modified embryos and their wild type controls, at least 3 embryos were stained per probe and genotype, showing highly reproducible patterns. RNA probes have been previously described: *Msgn1* [111]; *Sox2*, *Tbxt* and *Uncx4.1* [76]; *Shh* and *Cdx2* [110]; *Wnt5a* [112] and Fgf4 [113]. A probe for *Nr2f2* was prepared by amplifying a cDNA fragment by RT-PCR (primers provided below, Table 4) from total RNA isolated from E9.5 embryos with Tri Reagent (Sigma #93,289) according to the manufacturer’s protocol, and cloning it into pKS-bluescript. Stained embryos were included in 0.45% gelatin (Merck, 104,078), 27% bovine serum albumin (Roche, 9048–46-8) and 18% sucrose (Sigma, S0389) in PBS and then polymerized with 1.75% glutaraldehyde (Biochem Chemopharma, 507,130,500) and sectioned at 35 µm with a vibratome (Leica, VT1000S).

**Table 4:** Primers used to amplify in situ probes and used in RT-qPCR

Nr2f2	Fw	ACGAATTCTGCATGCAGCCTAACAACATC
	Rv	ATGGATCCATTGCTCTATGACTGAGGAGG
Porcn	Fw	TCCTTCCACAGCTACCTACAG
	Rv	ACACAAGTGGACAGTACAAGG
Wnt5a	Fw	CCATGTCTTCCAAGTTCTTCC
	Rv	TACTTCTGACATCTGAACAGG
β-Actin	Fw	TCTGGTGGTACCACCATGTAC
	Rv	TACTTGCGCTCAGGAGGAGC

### RT-qPCR

For Porcn quantification, total RNA was extracted from the posterior epiblast of wild type embryos at E7.5, E8.5 and E9.5 using Tri Reagent. For Wnt5a quantification, total RNA was extracted from the caudal region of wild type and *Wnt5a*^*∆CR1/∆CR1*^ embryos at E9.5 and E10.5 using Tri Reagent. 1 µg of RNA was used for reverse transcription into complementary DNA (cDNA) using NZY Reverse Transcriptase enzyme (NZYTech #MB124) and random hexamer mix (NZYTech #MB12901) following the manufacturer’s protocol. Real-time qPCR was performed in a QuantStudio 7 Flex real-time PCR system (Thermo Fisher) using iQ SYBR Green Supermix (Bio-rad #1,708,880) according to manufacturer’s instructions. Primers used are listed in Table 4. Quantification was determined using the standard curve method, and expression levels normalized to *β-Actin*. Statistical significance was assessed using Tukey’s multiple comparison test in Porcn quantification and unpaired t-test in Wnt5a quantification.

### Supplementary Information

**Additional file 1: Fig. S1.** Principal Component Analyses of RNA-seq and ATAC-seq data. **Fig. S3.** *Porcn* expression is downregulated in the posterior epiblast. **Fig. S4.** Sequencing data and embryonic image of wild type and *Raldh2*^*-/-*^ mutants. **Fig. S5.** Transgenic reporter analysis for CR2b lacking the specified TF binding sites. **Table S2.** RT-qPCR data values of *Porcn* expression normalized to *β-Actin. ***Table S4.** RT-qPCR data values of *Wnt5a* expression normalized to *β-Actin.*

**Additional file 2: Table S1.** Complete differential analysis results of RNA-seq datasets.

**Additional file 3: Fig. S2.** Protein-protein interaction network generated with the differentially expressed genes.

**Additional file 4: Table S3.** Complete differential analysis results of ATAC-seq E7.5 and E8.5 wild type datasets.

**Additional file 5: Table S5**. Complete differential analysis results of ATAC-seq wild type and *Raldh2*^*-/-*^ datasets.

## Data Availability

All data generated or analyzed during this study are included in this published article and its supplementary information files. The RNA-seq and ATAC-seq data have been submitted to the NCBI Gene Expression Omnibus (GEO accession numbers GSE220246 [114] and GSE220245 [115], respectively). All materials used in this work are available upon request.

## Abbreviations

ATAC-seq: Assay for Transposase-Accessible Chromatin with sequencing
CR: Conserved Region
CRISPR: Clustered Regularly Interspaced Short Palindromic Repeats
DEG: Differentially Expressed Genes
DMSO: Dimethylsulfoxide
GO: Gene Ontology
NMC: Neuro Mesodermal Competent cells
PBS: Phosphate Buffered Saline
PFA: Paraformaldehyde
PPI: Protein–Protein Interaction
PSM: Presomitic Mesoderm
RA: Retinoic Acid
RAR: Retinoic Acid Receptor
RARE: Retinoic Acid Responsive Element
RT-PCR: Reverse Transcriptase linked to Polymerase Chain Reaction
RXR: Retinoid X Receptor
TAD: Topologically Associating Domain
TF: Transcription Factor
