# Supplementary material for: Regulatory changes associated with the head to trunk developmental transition
Source: BMC Biol. 2023 Aug 8;21:170. doi: 10.1186/s12915-023-01675-2 (PMC10408190; doi:10.1186/s12915-023-01675-2)
Supplement: Supplementary file 1 — Additional file 1: Fig. S1. Principal Component Analyses of RNA-seq and ATAC-seq data. Fig. S3. Porcn expression is downregulated in the posterior epiblast. Fig. S4. Sequencing data and embryonic image of wild type and Raldh2-/- mutants. Fig. S5. Transgenic reporter analysis for CR2b lacking the specified TF binding sites. Table S2. RT-qPCR data values of Porcn expression normalized to β-Actin. Table S4. RT-qPCR data values of Wnt5a expression normalized to β-Actin. [file 12915_2023_1675_MOESM1_ESM.pdf]

Additional file 1

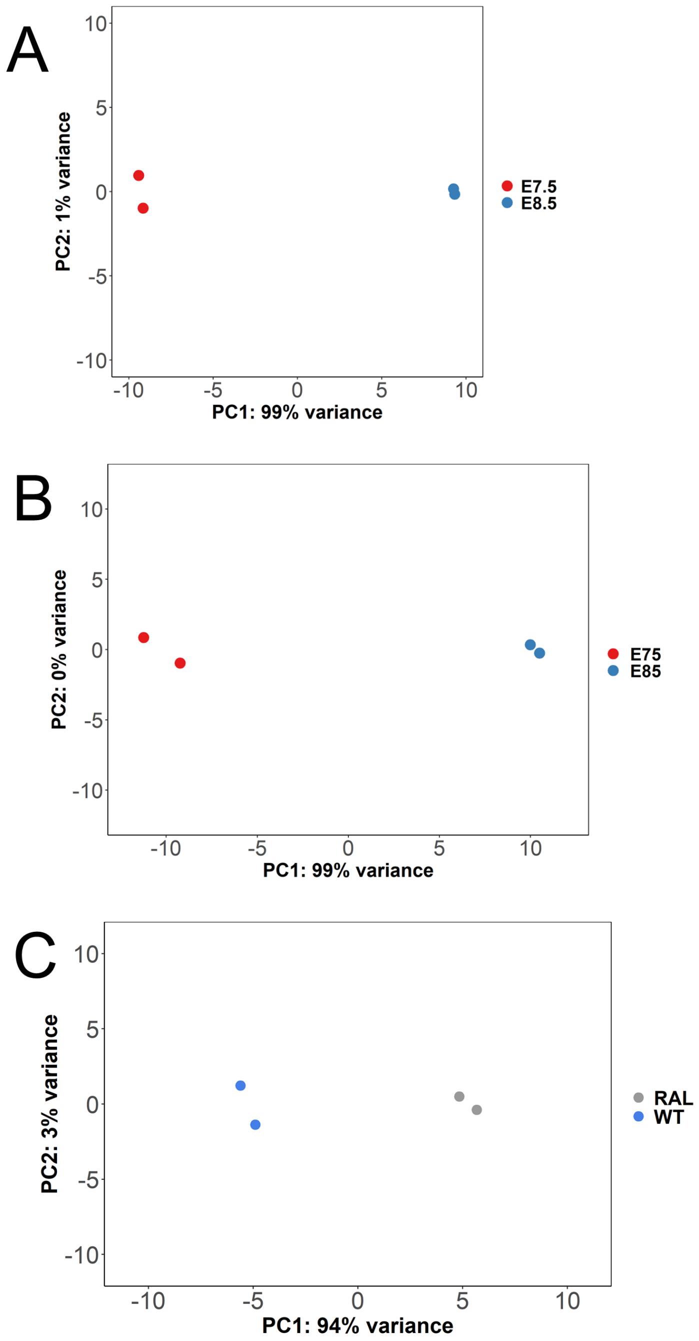

Figure S1 – (A) Principal Component Analysis of RNA-seq data from E7.5 (red) and E8.5 (blue). (B) Principal Component Analysis of ATAC-seq data from E7.5 (red) and E8.5 (blue). (D) Principal Component Analysis of ATAC-seq data from WT (blue) and *Raldh2*$^{-/-}$ (grey).

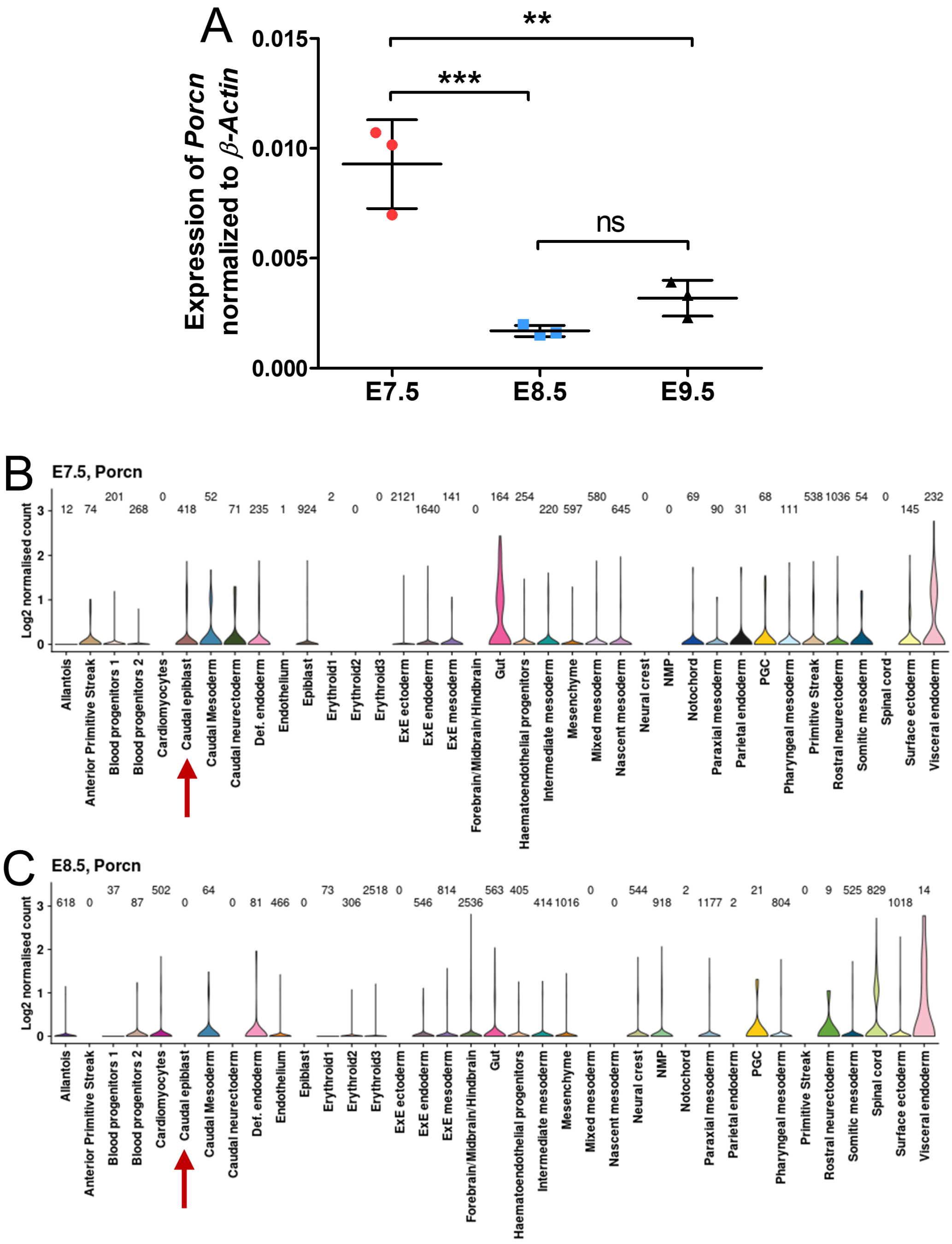

Figure S3 – *Porcn* expression is downregulated in the posterior epiblast. (A) RT-qPCR analysis of *Porcn* gene expression in this region of wild type embryos at E7.5, E8.5 and E9.5. *Porcn* expression was normalized to *β-Actin*. Error bars indicate the standard deviation; ***, p-value <0.001; **, p-value < 0.01 and ns, non-significant. (B-C) Violin plots showing Porcn expression levels at E7.5 (B) and E8.5 (C) from publicly available single-cell transcriptomic data [23]. Red arrows highlight the decrease in expression in the caudal epiblast cluster.

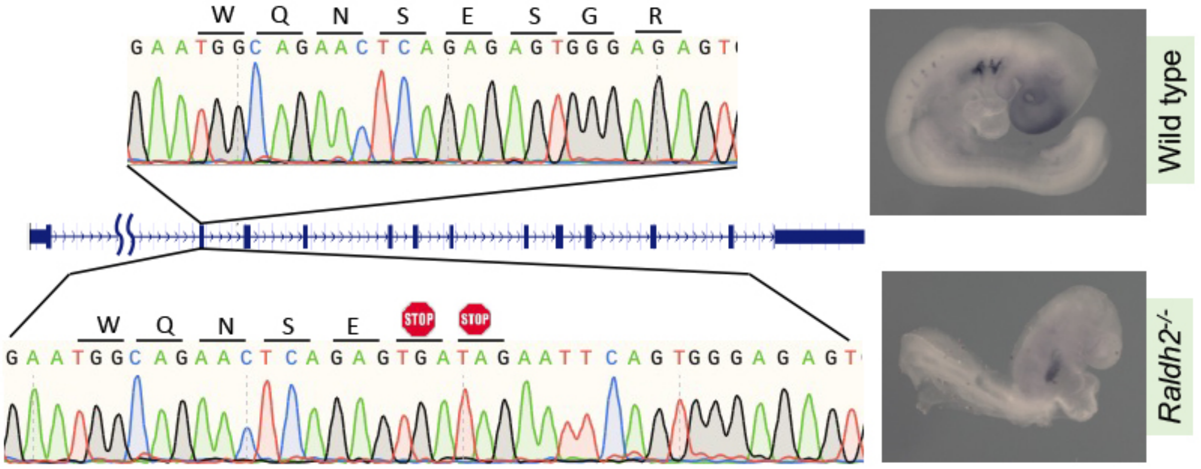

Figure S4 – Sequencing profiles of wild type and *Raldh2*$^{-/-}$ mutants, generated by introducing in frame stop codons in the second exon. Whole-mount in situ hybridization of wild type and *Raldh2*$^{-/-}$ mutant embryos at E9.5 using a probe for *Fgf4*.

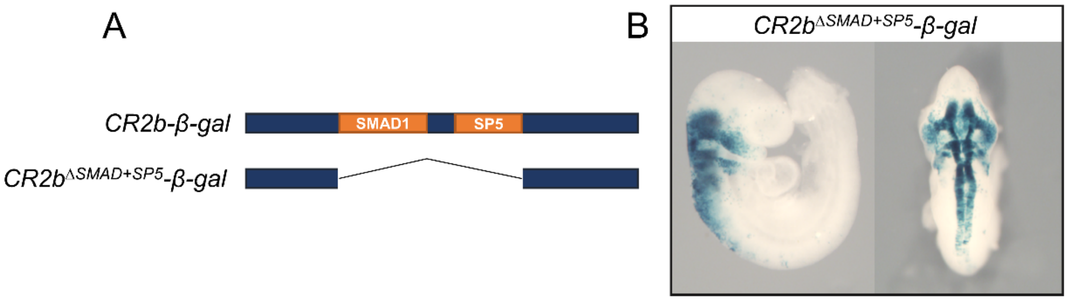

Figure S5– (A) Schematic representation of generated transgenic reporters for CR2b lacking the specified TF binding sites. (B) *β-gal* reporter expression in *CR2b*$^{\Delta SMAD1+SP5}$*-β-gal* transgenics (n=9/9), lateral and dorsal views.

Table S2. RT-qPCR data values of *Porcn* expression normalized to *β-Actin.*

	Expression of *Porcn* normalized to *β-actin*		
E7.5	0.00697826	0.01015979	0.01071465
E8.5	0.00198093	0.00160101	0.00150258
E9.5	0.00230666	0.00332555	0.00391798

Table S4. RT-qPCR data values of *Wnt5a* expression normalized to *β-Actin.*

	Expression of *Wnt5a* normalized to *β-actin*		
E9.5 WT	0.01868362	0.01757676	0.01922318
E9.5 KO	0.01077618	0.00907386	0.00921731
E10.5 WT	0.02573361	0.02787613	0.02689651
E10.5 KO	0.01931569	0.01569855	0.01985432
