# Supplementary figures and images for: Regulatory changes associated with the head to trunk developmental transition
Source: BMC Biol. 2023 Aug 8;21:170. doi: 10.1186/s12915-023-01675-2 (PMC10408190; doi:10.1186/s12915-023-01675-2)

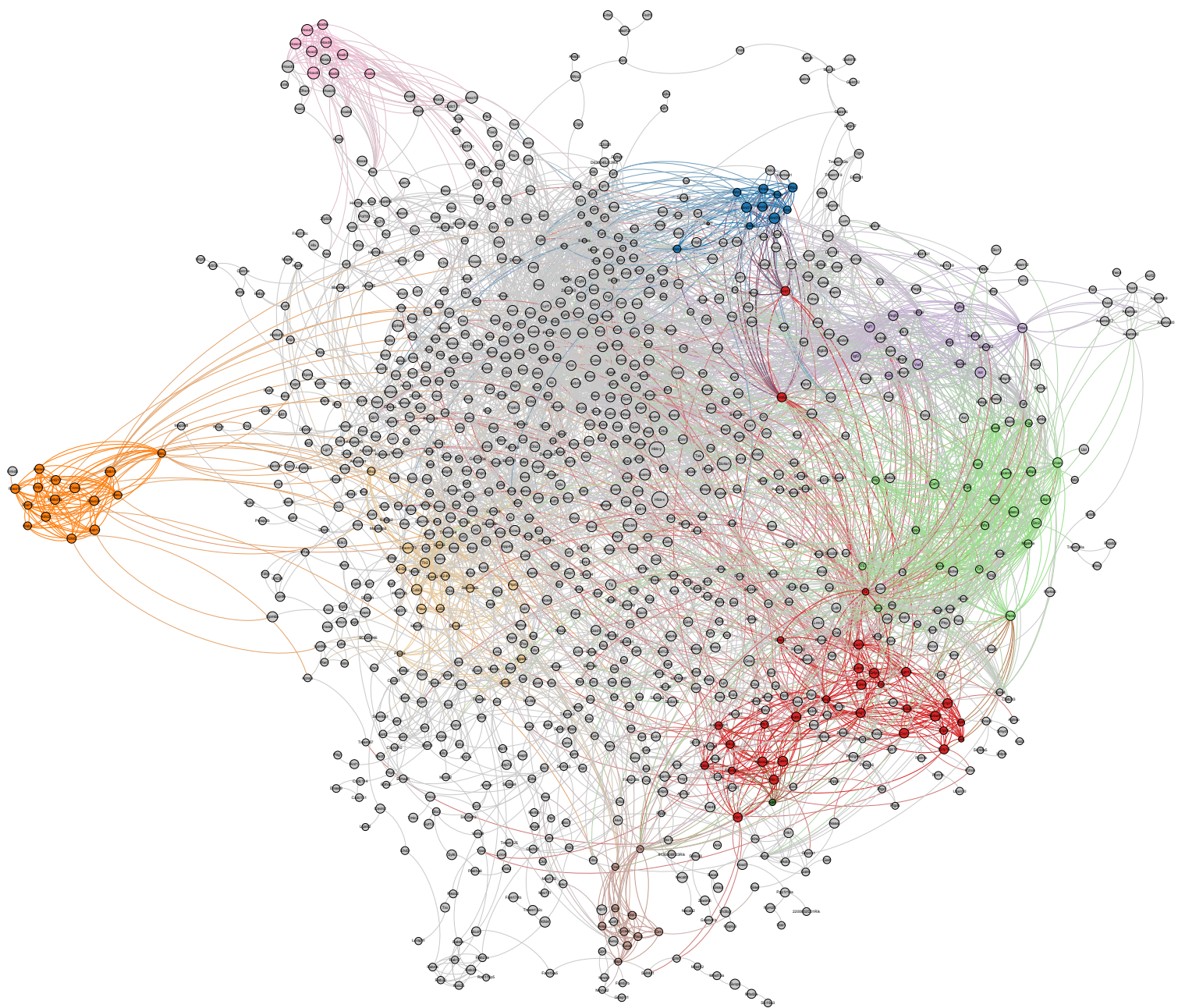

Supplement: Supplementary file 3 — Additional file 3: Fig. S2. Protein-protein interaction network generated with the differentially expressed genes. [file 12915_2023_1675_MOESM3_ESM.pdf]
